# A Comprehensive Evaluation of Nasal and Bronchial Cytokines and Chemokines Following Experimental Rhinovirus Infection in Allergic Asthma: Increased Interferons (IFN-γ and IFN-λ) and Type 2 Inflammation (IL-5 and IL-13)

**DOI:** 10.1016/j.ebiom.2017.03.033

**Published:** 2017-03-28

**Authors:** Trevor T. Hansel, Tanushree Tunstall, Maria-Belen Trujillo-Torralbo, Betty Shamji, Ajerico del-Rosario, Jaideep Dhariwal, Paul D.W. Kirk, Michael P.H. Stumpf, Jens Koopmann, Aurica Telcian, Julia Aniscenko, Leila Gogsadze, Eteri Bakhsoliani, Luminita Stanciu, Nathan Bartlett, Michael Edwards, Ross Walton, Patrick Mallia, Toby M. Hunt, Trevor L. Hunt, Duncan G. Hunt, John Westwick, Matthew Edwards, Onn Min Kon, David J. Jackson, Sebastian L. Johnston

**Affiliations:** a Airway Disease Infection Section, National Heart and Lung Institute (NHLI), Imperial College (IC), London, UK; b MRC & Asthma UK Centre in Allergic Mechanisms of Asthma, UK; c Imperial College Healthcare NHS Trust, UK; d Imperial Clinical Respiratory Research Unit (ICRRU), UK; e Novartis Institute for Biomedical Research, Horsham, UK; f MRC Biostatistics Unit, Cambridge Institute of Public Health, Cambridge, UK; g Dept. of Theoretical Systems Biology at IC, UK; h Medimmune, Cambridge, UK; i Hunt Developments (UK) Ltd, Midhurst, West Sussex, UK; j Guy's and St Thomas' NHS Trust

**Keywords:** Rhinovirus, Asthma, Mucosal immunology, Absorption of mucosal lining fluid, Interferons, Type II inflammation

## Abstract

**Background:**

Rhinovirus infection is a major cause of asthma exacerbations.

**Objectives:**

We studied nasal and bronchial mucosal inflammatory responses during experimental rhinovirus-induced asthma exacerbations.

**Methods:**

We used nasosorption on days 0, 2–5 and 7 and bronchosorption at baseline and day 4 to sample mucosal lining fluid to investigate airway mucosal responses to rhinovirus infection in patients with allergic asthma (*n* = 28) and healthy non-atopic controls (*n* = 11), by using a synthetic absorptive matrix and measuring levels of 34 cytokines and chemokines using a sensitive multiplex assay.

**Results:**

Following rhinovirus infection asthmatics developed more upper and lower respiratory symptoms and lower peak expiratory flows compared to controls (all *P* < 0.05). Asthmatics also developed higher nasal lining fluid levels of an anti-viral pathway (including IFN-γ, IFN-λ/IL-29, CXCL11/ITAC, CXCL10/IP10 and IL-15) and a type 2 inflammatory pathway (IL-4, IL-5, IL-13, CCL17/TARC, CCL11/eotaxin, CCL26/eotaxin-3) (area under curve day 0–7, all *P* < 0.05). Nasal IL-5 and IL-13 were higher in asthmatics at day 0 (*P* < 0.01) and levels increased by days 3 and 4 (*P* < 0.01). A hierarchical correlation matrix of 24 nasal lining fluid cytokine and chemokine levels over 7 days demonstrated expression of distinct interferon-related and type 2 pathways in asthmatics. In asthmatics IFN-γ, CXCL10/IP10, CXCL11/ITAC, IL-15 and IL-5 increased in bronchial lining fluid following viral infection (all *P* < 0.05).

**Conclusions:**

Precision sampling of mucosal lining fluid identifies robust interferon and type 2 responses in the upper and lower airways of asthmatics during an asthma exacerbation. Nasosorption and bronchosorption have potential to define asthma endotypes in stable disease and at exacerbation.

## Highlights

• Following rhinovirus infection asthmatics have increased interferons and type 2 inflammation in airway mucosal lining fluid.
• Nasosorption cytokines and chemokines showed distinct pathways of interferon and type 2 inflammation in asthma.
• Precision mucosal sampling has potential for stratifying molecular endotypes of asthma.
• Validation of nasosorption and bronchosorption will be required for selection of asthmatics for therapy with biologics.

Experimental human rhinovirus (HRV) infection causes more severe upper and lower respiratory tract symptoms in allergic asthmatics than in healthy controls. There is greater induction of cytokines and chemokines in nasal and bronchial mucosal lining fluid (MLF) of asthmatics: with distinct pathways of type 2 and anti-viral/regulatory inflammation.

Subject to further validation, analysis of MLF may prove useful in stratification of patients with asthma, and the definition of molecular endotypes.

**Interpretation**

Nasosorption and bronchosorption are precision sampling methods with potential for widespread application in respiratory and other mucosal diseases (*e.g.* gastrointestinal diseases).

Biomarkers identified in nasosorption and bronchosorption samples will need to be validated compared to established airway sampling methods, in a range of asthma phenotypes, and with current and novel therapies.

## 1 Introduction

Throughout life the human immune system is modulated by interactions with viruses, bacteria, and allergens (Hansel et al., 2013). Abnormal airway mucosal inflammatory responses are a feature of asthma, and an expanding number of asthma phenotypes are now recognised at a clinical and molecular level (Wenzel, 2012). Since rhinoviruses are a major cause of exacerbations of asthma (Jackson & Johnston, 2010), it is important to monitor rhinovirus-induced mucosal immune responses.

Airway inflammation and asthma phenotypes have been defined by sputum eosinophil and neutrophil percentages (Moore et al., 2014), and bronchial and nasal mucosal gene expression can be used to define endotypes of asthma (Choy et al., 2011, Poole et al., 2014). However, blood eosinophil counts are generally used to monitor inflammation in severe asthma, although there is recognition of the need to develop non-invasive sampling methods coupled to assessment of biomarkers (Chung et al., 2014). Based on the presence of airway eosinophilia and type 2 inflammation in asthma (Choy et al., 2011, McGrath et al., 2012, Fahy, 2015), biologic therapies targeting type 2 inflammation are being introduced for selected asthmatics (Hambly and Nair, 2014, Chung, 2015).

The magnitude of interferon production in asthma remains controversial. Deficiency of various interferons (IFNs) has been demonstrated using cultured human bronchial epithelial cells (hBECs) from asthmatics following rhinovirus infection *in vitro*. This includes deficiency of IFN-β and IFN-λ in primary bronchial epithelial cells (PBECs) (Wark et al., 2005, Contoli et al., 2006, Edwards et al., 2013), and deficiency of IFNs (γ, α, β and λ) and IL-15 in BAL cells (Contoli et al., 2006, Message et al., 2008, Sykes et al., 2012, Laza-Stanca et al., 2011). However, rhinovirus-induced interferon production in hBECs is not deficient in well-controlled asthma (Sykes et al., 2014), hBECs from asthmatics have preserved interferon responses to influenza and respiratory syncytial virus (RSV) (Patel et al., 2014), and TLR responses are not impaired in asthmatic airway and blood cells (Sykes et al., 2013). In addition, robust IFN-γ and IFN-λ responses have been found clinically in children with asthma during naturally-occurring virus-induced exacerbations (Lewis et al., 2012, Miller et al., 2012), and severe adult asthmatics and neutrophilic asthmatics have recently been shown to have a dominant IFN-γ immune response in BAL (Raundhal et al., 2015, da Silva et al., 2017).

Nasosorption with paper and then synthetic absorptive matrices (SAM) has been used to sample nasal mucosal lining fluid (Alam et al., 1992, Lu and Esch, 2010, Scadding et al., 2012, Jochems et al., 2017). This technique can be considered as “precision mucosal sampling”, since it samples directly from the respiratory mucosa, and is free from the salivary contamination that occurs in breath and sputum sampling. Nasosorption sampling involves manipulating the synthetic absorptive matrix (SAM) up the lumen of the nasal cavity, and then holding it in position against the mucosa by external firm finger pressure. This is more comfortable and less invasive than using a conventional swab, where rotation against the mucosal surface is generally required.

Sampling by nasosorption has been carried out in children with rhinitis (Chawes et al., 2010), neonates (Folsgaard et al., 2012), and after nasal allergen challenge (Scadding et al., 2012, Scadding et al., 2015, Nicholson et al., 2011, Leaker et al., 2017). Nasal lavage causes considerable dilution of nasal mediators, resulting in levels approximately tenfold less than those found in nasosorption samples (Riechelmann et al., 2003, Hentschel et al., 2014). The absorption technique of bronchial microsampling has also been developed (Ishizaka et al., 2001), but this sampling method caused mucosal bleeding in patients with asthma (Cohen et al., 2008), and we have developed a modified bronchosorption system.

This study involves experimental rhinovirus infection followed by serial nasosorption and bronchosorption sampling, with the aim of studying upper and lower airway mucosal immune responses in individual patients with asthma. Following experimental rhinovirus infection we have previously reported baseline and peak nasal responses, together with baseline and day 4 bronchial responses of IL-4, IL-5, IL-13 and IL-33 (Jackson et al., 2014), IL-15 (Jayaraman et al., 2014); IL-18 (Jackson et al., 2015), and IL-25 (Beale et al., 2014) in samples from the current study. In this report we provide a more complete presentation of levels of 34 cytokines and chemokines from the nose and lung, based on presentation of values recorded at defined time points in individual patients.

## 2 Materials and Methods

### 2.1 Study Participants

Non-smoking asthmatic and healthy non-asthmatic volunteers aged 18–55 years with absent serum neutralising antibodies to rhinovirus-16 (RV16) were recruited (Jackson et al., 2014).

### 2.2 Study Approval

This clinical study received Research Ethics Committee approval (09/H0712/59) and written informed consent was obtained from all participants prior to inclusion in the study.

### 2.3 Inclusion and Exclusion Criteria

Asthmatic subjects were eligible for inclusion if they had a doctor diagnosis of asthma and objective evidence of airway hyperresponsiveness with a (PC)_20_ histamine < 8 μg/mL, evidence of atopy on skin prick testing (≥ 1 positive skin prick test on a panel of 10 common aeroallergens), and a history of worsening asthma symptoms with infection (Jackson et al., 2014). Subjects were excluded if they had a history of severe asthma or any significant other respiratory or medical disease, smoking of > 5 pack year history or any smoking within the previous 6 months, current symptoms of allergic rhinitis, or had experienced an asthma exacerbation or viral illness within the previous 6 weeks.

Healthy, non-smoking subjects were eligible if they had no history of asthma, allergy or any other significant disease, had negative skin prick testing, and no objective airway hyperresponsiveness with a histamine (PC)_20_ ≥ 8 μg/mL (Jackson et al., 2014).

Pregnant or breastfeeding women and any subject who had contact with infants or the elderly at home or at work were excluded. Further exclusion criteria included use of any nasal or oral medication including anti-histamines, or nasal/oral corticosteroids. Subjects were excluded from the study analysis if they failed to develop objective evidence of a rhinovirus infection defined as serum neutralising antibody to RV16 at 6 weeks of < 1:4 and no detection of RV16 in nasal lavage samples throughout the infection period (day 2–10) (Jackson et al., 2014).

### 2.4 Study Design

**Fig. 1:**
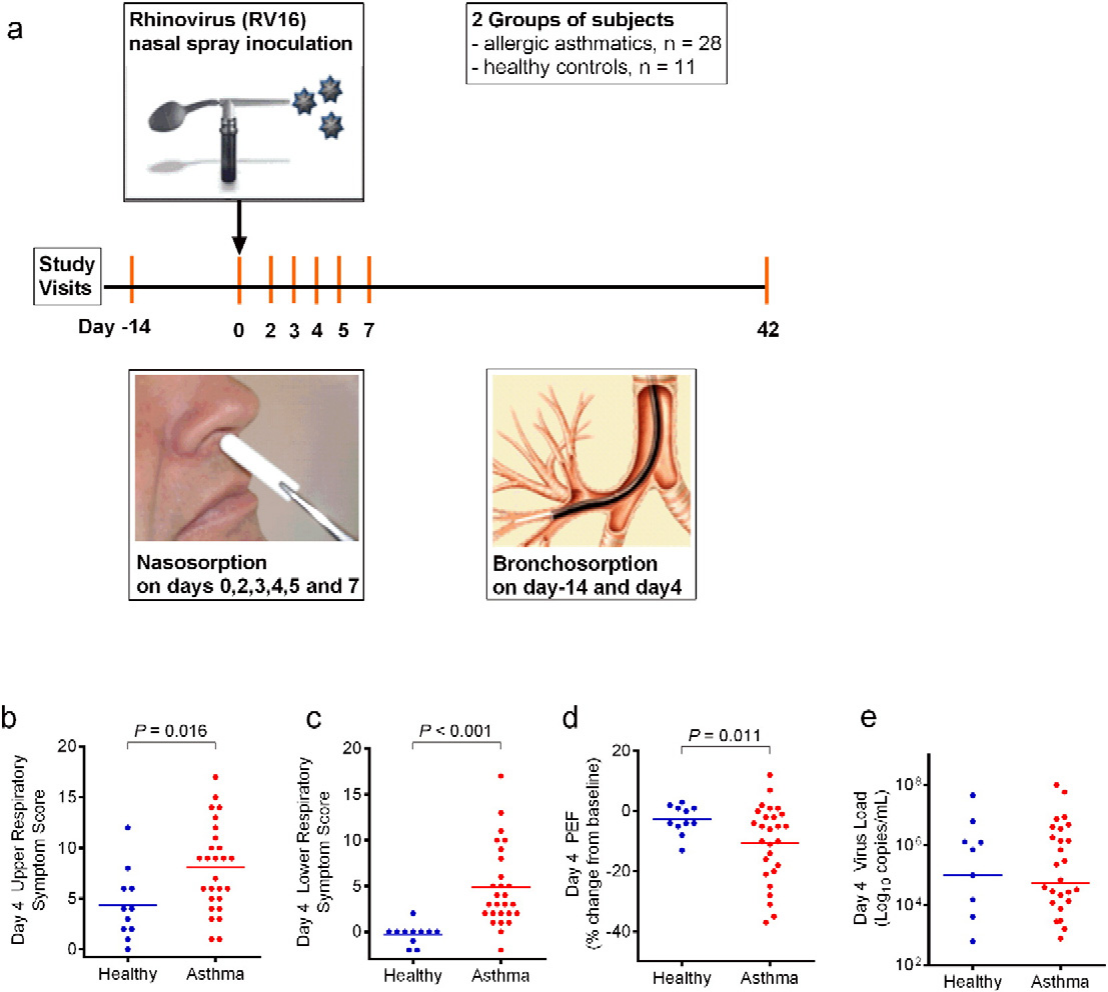
Study design with clinical and viral load responses to rhinovirus infection. Nasal inoculation with rhinovirus 16 (RV16) was performed in 28 asthmatic and 11 healthy controls (a). Nasal mucosal lining fluid was sampled by the technique of nasosorption on days 0,2,3,4,5 and 7 post-inoculation. Bronchial lining fluid was sampled by bronchosorption at baseline (− 14d) and day 4 post-inoculation. Upper (b) and lower (c) respiratory tract symptom scores, with changes in morning peak expiratory flow (PEF) from baseline (d) and nasal viral load (e) are shown for asthmatic (red) and healthy (blue) subjects. Bars represent mean values (b-d) or median values (e). This clinical data and the viral load responses have previously been reported (Jackson et al., 2014).

Volunteers underwent bronchoscopy with bronchosorption ~ 14 days prior to inoculation with RV16 and on day 4 (Fig. 1A). Nasosorption was performed on days 0, 2–5 and 7. On day 0, nasosorption and then nasal lavage sampling was performed immediately before inoculation with virus (HRV16). RV16 (Bardin et al., 1996) was inoculated into both nostrils (total dose 100 TCID_50_) using an atomizer (no. 286; De Vilbiss Co., Heston, UK) on day 0. Rhinovirus was detected during the infection by PCR of nasal lavage (Scadding et al., 2015). Daily diary cards of respiratory symptoms (Message et al., 2008) were commenced 2 weeks prior to baseline sampling and continued until 6 weeks after inoculation, with lower respiratory symptom scores corrected for the effects of bronchoscopy (Message et al., 2008, Jackson et al., 2014). Spirometry was performed on waking using a Piko-1 spirometer (nSpire Health, CO).

### 2.5 Nasosorption

Nasosorption was performed by placing strips of a hydrophilic polyester absorptive matrix (Mucosal Diagnostics, Hunt Developments (UK) Ltd., Midhurst, UK: available as a CE-marked device) measuring 7 × 35 mm into each nostril for 2 min (Jackson et al., 2014). Having removed the SAM strip, it was washed in PBS buffer pH 7.4 (100 μl) containing BSA (1%) and Triton X 100 (1%) within the cup of a spin filter insert (Costar® Spin-X®). Mucosal lining fluid was then eluted from the SAM by spin filter centrifugation (5 min at 16,000G at 4 °C), and aliquots frozen at − 80 °C.

### 2.6 Bronchosorption

The bronchosorption catheter was passed down the operating port of a bronchoscope, and has an inner SAM probe that is extruded under direct vision from the catheter using a handpiece (Hunt Developments (UK) Ltd., Midhurst, UK: available as a CE-marked device). The main benefit of bronchosorption sampling is the detection of relatively high levels of mediators through avoiding the significant and variable analyte dilution associated with bronchoalveolar lavage. An inner probe wire is tipped by a SAM (1.8 × 30 mm) which was placed on the bronchial mucosa for a period of 30 s. Following sampling, the bronchosorption device was withdrawn back into its catheter, and the complete device was removed from the bronchoscope. The sampling end of the probe was then cut off and treated in an identical way to the nasosorption strips.

### 2.7 Cytokine and Chemokine Immunoassays

Levels of 34 cytokines and chemokines in the extracted upper and lower airway mucosal lining fluid were analysed using ultrasensitive Meso Scale Discovery multi-spot human cytokine assays (Meso Scale Discovery, Gaithersburg, MD, USA). Samples were read using the Sector Imager 6000 (Meso Scale Discovery).

### 2.8 Multiplex Immunoassay of Cytokines and Chemokines

The 34 cytokines and chemokines measured were IL-1β, IL-2, IL-4, IL-5, IL-6, IL-10, IL-12p40, IL-12p70, IL-13, IL-15, IL-16, IL-17, IL-18, IL-25, IL-29 /IFN-λ, IL-33, IFN-β, IFN-γ, TSLP, TNF-α, GM-CSF, CCL2/MCP1, CCL3/MIP1α, CCL4/MIP1β, CCL5/RANTES, CCL11/eotaxin, CCL13/MCP4, CCL17/TARC, CCL20/MIP3α, CCL22/MDC, CCL26/eotaxin3, CXCL8/IL-8, CXCL10/IP10, CXCL11/ITAC. The lower limit of detection was 1.0 pg/ml for all mediators except CCL26/eotaxin-3, CCL2/MCP-1, CCL13/MCP-4 and IL-33 (3 pg/ml); IL-16 (5 pg/ml); CCL11/eotaxin, CXCL10/IP10, CCL17/TARC, IL-25, TSLP and CCL4/MIP1β (10 pg/ml); IFN-β (25 pg/ml); IL-29/IFN-λ (40 pg/ml) and CCL22/MDC (100 pg/ml).

### 2.9 Statistical Analysis and Data Processing

Mucosal lining fluid eluate levels of cytokines and chemokines were not normally distributed (Shapiro-Wilks test). Statistical analysis was carried out for paired samples using the Wilcoxon signed-rank test, and for non-paired samples with the Mann-Whitney test.

A Dynamical Hierarchical Correlation Matrix was produced for nasosorption levels of 24 cytokines and chemokines on days 0, 2–5 and 7. Hierarchical dynamical correlation between the longitudinal cytokine/chemokine profiles of individuals within each group is represented as a heat map with cytokine clustering (Opgen-Rhein & Strimmer, 2006), using R statistical software (version 2.15.2).

A Volcano Plot was performed on normal transformed data for all 34 cytokines and chemokines, assessing the fold change between asthmatics and controls from day 0 in relation to days 2–5 and 7 post infection. The fold change was calculated between asthmatics and controls using mean levels of cytokine or chemokine on particular days. The *P* value was derived by *t*-test and corrected for multiple testing (Benjamin-Hochberg). Cluster Analysis of nasosorption levels was carried out in relation to IFN-γ, IL-5 and IL-13 (Z score normalised) on day 0 and day 4.

## 3 Results

### 3.1 Study Subjects and Clinical Outcomes

28 allergic asthmatics and 11 healthy control subjects were successfully infected with RV16 (Jackson et al., 2014, Beale et al., 2014) (Table 1). Nasosorption and bronchosorption were well tolerated with no significant adverse events. Respiratory symptoms and lung function measurements were recorded daily throughout the study and have been previously reported (Jackson et al., 2014), but we highlight day 4 after infection (Fig. 1B, C, D), since bronchosorption was performed on this day. On day 4 nasal viral load was not significantly different between asthmatics and controls (Fig. 1E), although on day 3 viral load was significantly increased in asthmatics (Fig. S1) (Jackson et al., 2014). Virus load in asthmatics was not significantly increased when assessing area under the curve for days 2–7 (Mann Whitney test, *P* = 0.0833). Viral load on day 3 was assessed for correlation with levels of nasal cytokines on day 3 (Table S1), viral load on day 4 was assessed for correlation with levels of nasal cytokines on day 4 (Table S2), and also AUC for days 2–7 for both viral load and nasal cytokines were also analysed (Table S3).

**Table 1:** Baseline characteristics of study volunteers.

Characteristic	Healthy (*N* = 11)	Asthma (*N* = 28)	*P*
Age (yr)	31 ± 12	36 ± 11	*NS*
Sex (%)			
Female	4 (36)	15 (54)	
Male	7 (64)	13 (46)	*NS*
White ethnicity (% of subjects)	9 (82)	22 (79)	*NS*
Percent predicted FEV_1_	104 ± 8	86 ± 12	< 0.001
Histamine PC_20_ (mg/mL)	> 16	1.3 ± 2.0	–
Asthma control questionnaire (ACQ) score	–	1.1 ± 0.6	–
ICS use (%)	–	15 (54)	–
ICS daily dose Beclometasone equivalent (mcg) (mean of steroid-treated subjects, *n* = 15)	–	427 ± 71	–
IgE IU/mL, median (IQR)	16 (14–19)	139 (70–448)	< 0.001
Aeroallergen sensitivity (no. of positive skin prick tests)	–	2.7 ± 1.2	–
Blood eosinophils (per μL)	100 ± 63	264 ± 213	< 0.001

These baseline characteristics have been previously reported (Jackson et al., 2014, Beale et al., 2014).

Means ± standard deviation unless otherwise stated; FEV_1_ = forced expiratory volume in 1 s; ICS = inhaled corticosteroids; IQR = interquartile range, NS = not significant.

Threshold for significance is defined as *P* < 0.05.

### 3.2 Nasal Cytokines/Chemokines

We have previously reported that baseline nasal levels of IL-4, IL-5, IL-13 were significantly increased in these asthmatic subjects compared to controls (Jackson et al., 2014). IL-4, IL-5 and IL-13 become significantly increased in asthmatics on and off inhaled corticosteroids (ICS), and in both mild and moderately severe asthmatics.

**Fig. 2:**
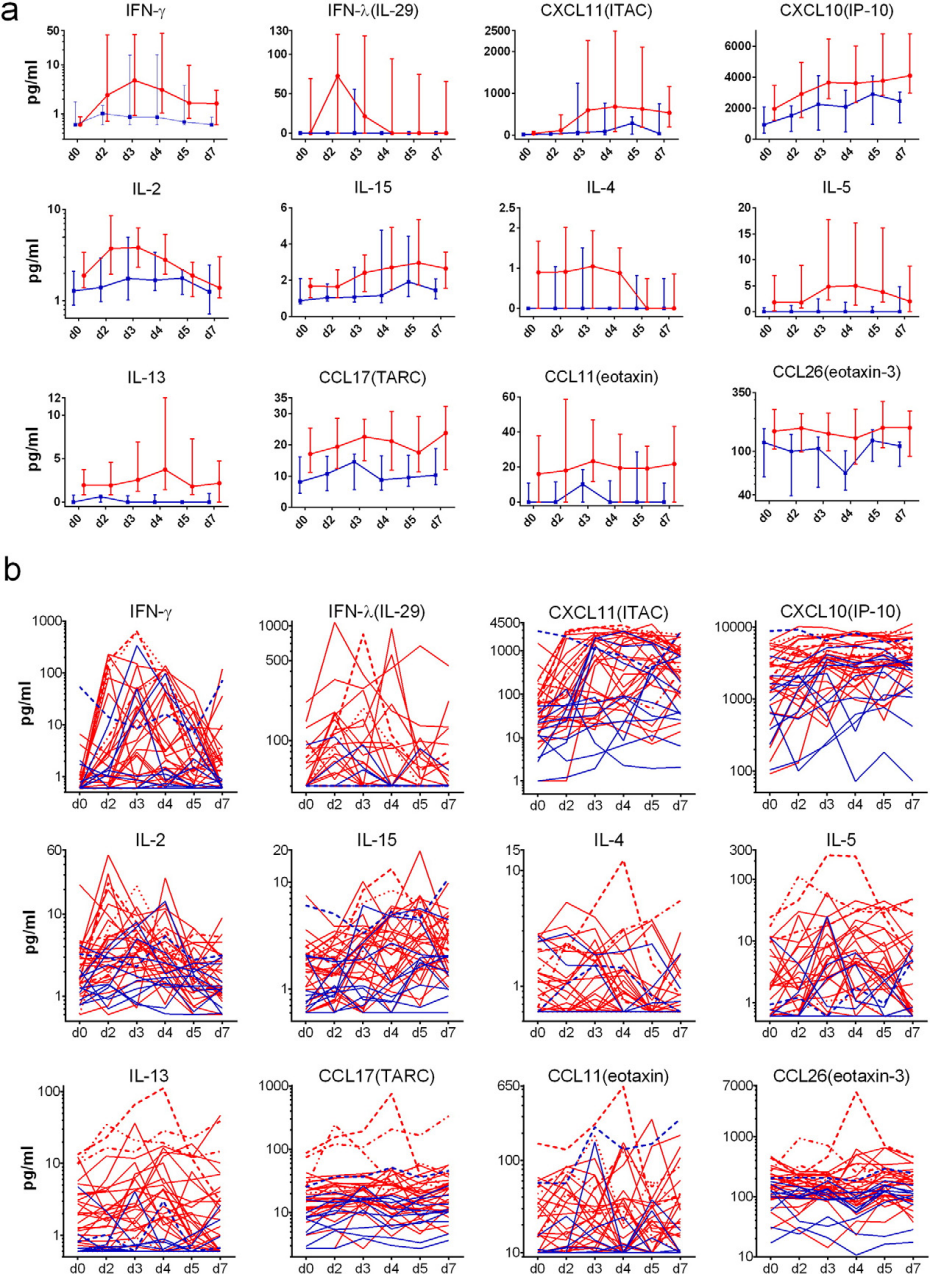
Nasal mucosal lining fluid cytokine and chemokine levels. Levels of 12 cytokines and chemokines in nasal mucosal lining fluid obtained by nasosorption were determined by multiplex immunoassay. (a). Median levels with quartiles for allergic asthmatics (red) and healthy control subjects (blue). (b). Individual values for all subjects (shown on log scale): asthmatics (red, *n* = 28) and healthy subjects (blue, *n* = 11). Four representative asthmatics and one healthy control subject are given specific line patterns to allow identification of these individuals across the panel of cytokine responses. See Tables S4–9 for relevant statistical analyses on nasosorption levels of cytokines and chemokines: noting that for the 12 cytokines and chemokines displayed the area under the curve (AUC) for days 0–7 was significantly greater in allergic asthmatics than in healthy controls (*P* < 0.05 in all cases) (Table S5).

We now report the type 2 chemokines CCL11/eotaxin, CCL17/TARC and CCL26/eotaxin-3 were also increased (*P* < 0.05) (Table S5). The levels of 34 cytokines and chemokines in nasal lining fluid were measured over the first week of the infection, noting that some levels have been reported largely as peak values (Jackson et al., 2014, Jackson et al., 2015, Jayaraman et al., 2014, Beale et al., 2014). Twelve nasal cytokines and chemokines were selected for presentation (Fig. 2) based on them having significantly (*P* < 0.05, AUC days 0–7) increased levels in asthmatics compared to controls (Table S4). IFN-β, CCL2/MCP-1 and CCL13/MCP-4 were also significantly increased, but are not presented here as levels were mostly undetectable [IFN-β] or for reasons of space [CCL2/MCP-1 and CCL13/MCP-4]. The remaining 22 cytokines and chemokines are presented in Figs. S2 and S5. Median levels with quartiles permit an assessment of the overall magnitude and kinetics of the response (Fig. 2A and Fig. S2). Actual levels for individuals over time are shown on logarithmic and linear scales, with levels for 5 individuals given markings for identification (Fig. 2B and Figs. S3–S4). Levels of IL-5 and IL-13 in nasal lining fluid tend to be high before infection (day 0) in those individuals that developed the highest levels after rhinovirus infection (Fig. S3).

Analysis of changes from baseline in healthy control and asthmatic subjects in nasal levels of all 34 cytokines/chemokines on days 2, 3, 4, 5 and 7 are given in Tables S5–S9 respectively. Responses to infection in the asthmatic subjects were remarkably robust, with 24 cytokines/chemokines being induced significantly on at least one day, with most reaching their maximum levels on days 3 to 5. However, when interpreting these responses, it should be emphasised that there were only 11 controls compared with 28 asthmatics. Prominent among those induced in asthma were cytokines of the dendritic cell/T cell axis including IL-2, IL-10 and CCL20/MIP3α, the type 2 pathway including IL-5, IL-13, IL-33, CCL11/eotaxin, CCL17/TARC and CCL22/MDC, the anti-viral interferon pathway including IFN-β, IFN-γ, IFN-λ/IL-29, IL-12p40, IL-15, CCL5/RANTES, CXCL10/IP10, CXCL11/ITAC and the pro-inflammatory cytokines TNFα, IL-6 and IL-17 and chemokines CCL2/MCP-1, CCL3/MIP-1α and CCL4/MIP-1β. IL-18 was lower on day 2 than at baseline in asthmatics and significantly lower in asthmatic compared to normal subjects on day 4 (Jackson et al., 2015) and IL-4 and CCL13/MCP-4 were also decreased after infection in asthmatics on days 5 and 7 and day 2 respectively (Tables S5 and S7–9). Cytokines not significantly induced on any post-infection day in asthma were IL-1β, IL-12p70, IL-16, IL-18, IL-25, GM-CSF, TSLP, CCL13/MCP-4, CCL26/eotaxin3 and CXCL8/IL-8 (Tables S5 and S6–9), though CCL13/MCP-4 was significantly increased after infection in asthma compared to healthy controls when assessing AUC (Table S4), suggesting greater data variability than with other cytokines/chemokines. Nasal mucosal cytokine and chemokine responses to infection in the healthy controls were smaller, with occasional significant increases only observed in IL-12p40, CCL17/TARC, CCL2/MCP-1, CCL22/MDC on days 2–5 and 7 (Tables S5, and S6–9).

### 3.3 Heat Map of Hierarchical Correlation Matrices

**Fig. 3:**
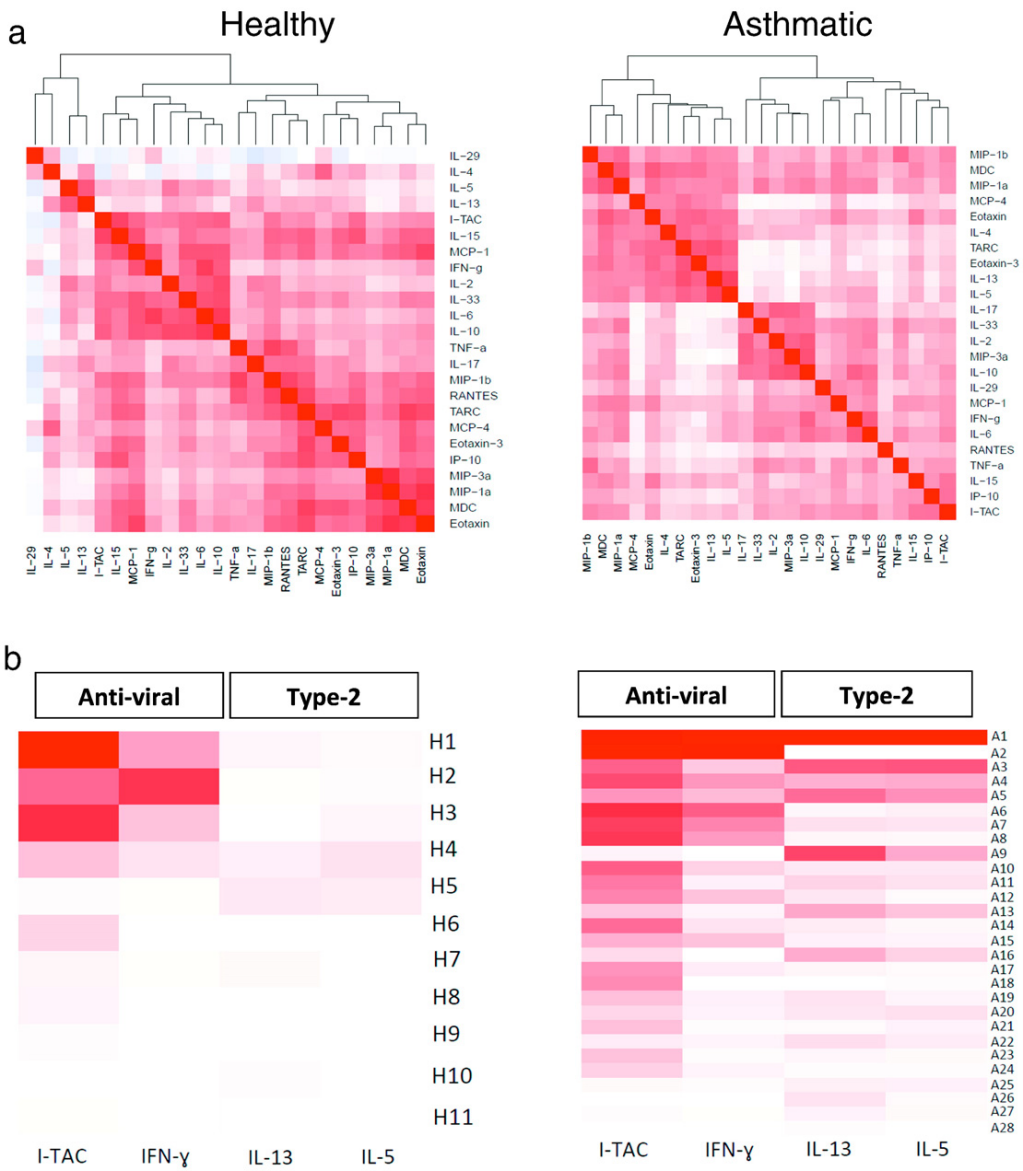
Nasal mucosal lining fluid heat maps. (a) Dynamical hierarchical correlation matrix for nasosorption levels of 24 cytokines and chemokines on days 0 to 7 generated using R statistical analysis software (version 2.15.2) (Opgen-Rhein & Strimmer, 2006). Cytokines are grouped together according to the strength of correlation using hierarchical clustering, as represented by the dendrogram. Positive or negative correlation is shown in the colour key. (b) Individual responses in terms of nasal cytokine and chemokine responses in individual subjects are shown as individual rows for separate healthy control (*n* = 11) (left) and allergic asthmatic (*n* = 28) (right) subjects. The order of asthmatic and healthy subjects was generated within a group in terms of the overall AUC (day 0–7) intensity, ranging from the highest mean value at the top to the lowest mean value at the bottom (controls and asthmatics together). The columns are for representative anti-viral (IFN-γ and ITAC/CXCL11) and type 2 (IL-5 and IL-13) cytokines and chemokines. R statistical analysis software (version 3.0.2) was used to generate heat maps. Colour intensity ranges from dark red (close correlation) to white (minimum correlation) for each cytokine/chemokine across all subjects. For 2 missing samples the last observation was carried forward (LOCF).

From the original 34 cytokines and chemokines measured in the nose we excluded TSLP, IL-1β, IL-12p40, IL-12p70, IL-16, IL-18, IL-25, IFN-β, GM-CSF and CXCL8/IL-8 where nasal levels were mostly below detection limits, and/or not significantly different in asthmatics *versus* controls (Table S4), or levels did not significantly increase from day 0 to day 4 (Table S7). A hierarchical correlation matrix was derived for nasal levels of the remaining 24 cytokines and chemokines, involving determination of incremental daily correlations beginning on day 0 and proceeding through days 2, 3, 4, 5 and then 7 (Fig. 3A).

In asthmatics (right) four major clusters can be seen:

• a small chemokine cluster containing CCL3/MIP-1α, CCL4/MIP-1β and CCL22/MDC.
• a clearly differentiated type 2 pathway including IL-4, IL-5, IL-13, CCL17/TARC, CCL11/eotaxin, CCL26/eotaxin-3 and CCL13/MCP-4.
• a mixed immune response cluster including IL-17, IL-33, IL-2, CCL20/MIP3α and IL-10.
• an interferon/inflammatory response cluster with IFN-γ, IFN-λ/IL-29, IL-6 and CCL2/MCP-1, CCL5/RANTES, TNFα, IL-15, CXCL10/IP10 and CXCL11/ITAC.

In the healthy controls (left) there is more fragmented clustering:

• type 2 inflammation (IL-5 and IL-13)
• anti-viral (CXCL11/ITAC, IL-15, CCL2/MCP-1, IFN-γ) with innate/regulatory factors (IL-2, IL-33, IL-6 and IL-10)
• pro-inflammatory cytokines (TNFα, IL-17) and a group of 10 chemokines.

### 3.4 Heat Map of Personalised Responses

This heat map demonstrates the size of the AUC for levels of particular nasal cytokines and chemokines in individual subjects, with a gradation of colour intensity set across all the asthmatics and controls for a given cytokine or chemokine (Fig. 3B). We have selected IFN-γ and ITAC/CXCL11 to reflect the anti-viral response, while IL-5 and IL-13 were chosen to represent type 2 inflammation. In the healthy controls (left) the nasal levels are lower, although 3 subjects had strong anti-viral responses (H1, H2, H3). As might be expected, IL-5 and IL-13 levels were conspicuously low in all the healthy subjects. In the asthmatics (right) a varied immune response (both qualitatively and quantitatively) was observed. Some asthmatics had strong interferon and IL-5/13 inflammatory responses (A1), others had strong anti-viral but weaker IL-5/13 responses (A2, A6–8), whilst others had a limited anti-viral response with a pronounced IL-5/13 response (A9).

### 3.5 Volcano Plot

**Fig. 4:**
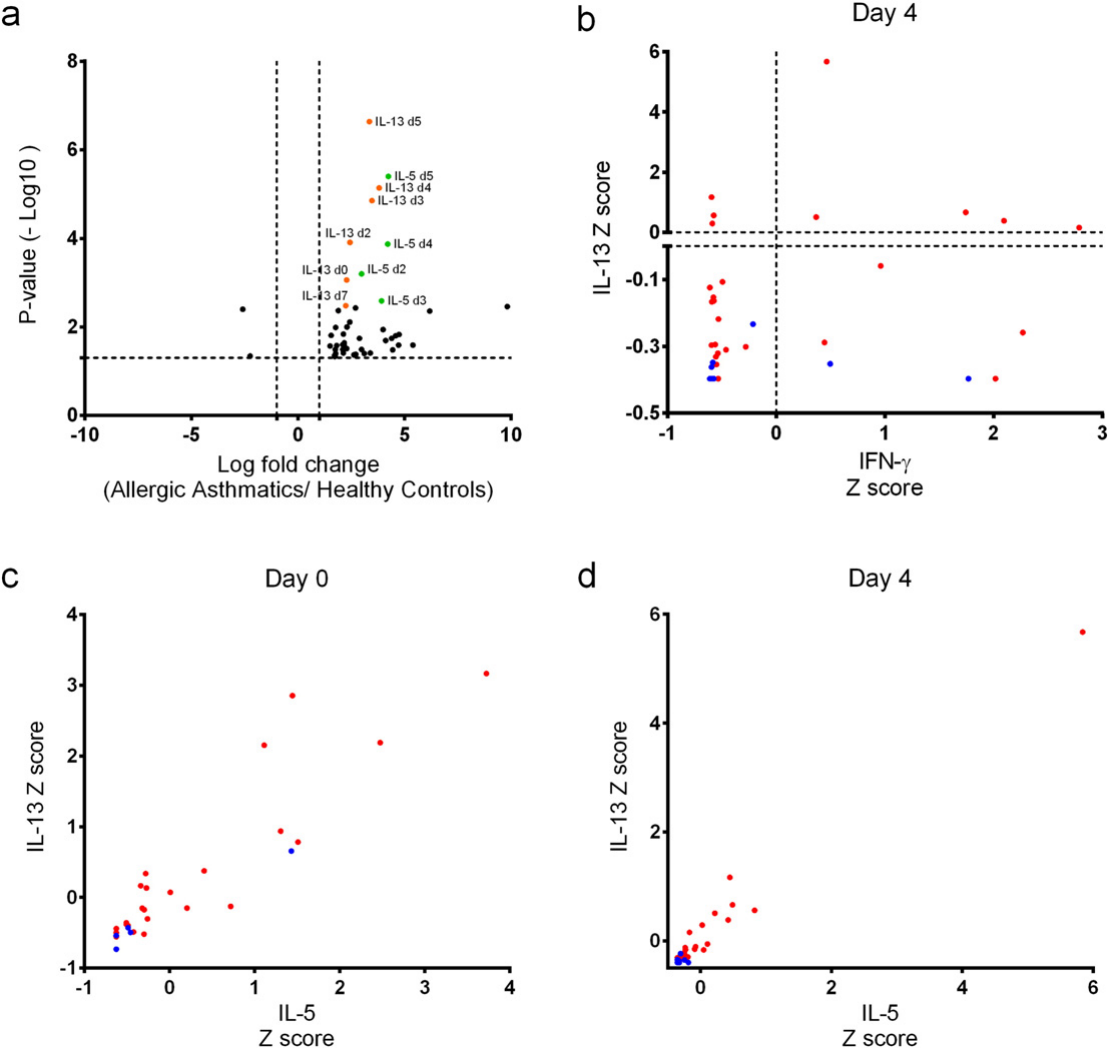
Nasal Mucosal Lining Fluid Volcano Plot and Cluster Analyses. (a) Volcano plot analysis was performed on normal transformed data for all 34 cytokines and chemokines, assessing the fold change between asthmatics and controls from day 0 in relation to days 2,3,4,5 and 7 post infection. The fold change was calculated between asthmatics and controls using mean levels of cytokine or chemokine on particular days. *P* values were derived by *t*-test and corrected for multiple testing (Benjamin-Hochberg), the horizontal dotted line demonstrates the cut off value for *P* = 0.05. (b) Cluster analysis of nasosorption levels of IFN-γ and IL-13 on day 4, with horizontal and vertical lines on zero. This shows the distribution of asthmatic subjects (red dots) and healthy controls (blue): asthmatics have high and low levels of IFN-γ and IL-13 (with red dots in 4 quadrants), while control subjects have low levels of IL-13 but can have higher levels of IFN-γ (with blue dots in 2 quadrants). (c) (d) These cluster analyses used levels of nasosorption IL-5 and IL-13 (Z score normalised) on day 0 and day 4 plotted as a 2D cluster. This illustrates how nasal IL-5 and IL-13 can be used to discriminate asthmatics from controls, and how nasal IL-5 and IL-13 levels are related.

A volcano plot of normalised data shows that asthmatics have a greater immune response as shown by the up-regulation of many cytokines, since the volcano plot is highly asymmetric to the right. In particular, nasal IL-5 and IL-13 are upregulated in asthma *versus* controls, especially on days 3–5 (Fig. 4A), and this confirms the greater induction demonstrated in terms of changes from baseline in asthmatic compare to healthy subjects (Table S10).

### 3.6 Receiver Operating Characteristic (ROC) Curves

The ROC curves presented in Fig. S6 and Table S11 display how nasosorption eluate IL-13 levels on days 0 and 4 can discriminate asthmatics and controls (AUCs 0.75 and 0.84 respectively).

### 3.7 Cluster Analyses

In considering the response to viral infection on day 4, we have previously noted that discrete pathways of interferon family and type 2 pathways are induced in nasal lining fluid (Fig. 3A). Nasal IL-5 and IL-13 are prominent in asthmatics (Fig. 4A). We then selected IFN-γ and IL-13 as representative members of interferon and type 2 pathways, to compare the viral response in asthmatics (red) and controls (blue) on day 4 (Fig. 4B). The viral response can be divided into quartiles based on high and low IL-13 and IFN-γ levels. This demonstrates the lack of relationship between IL-13 and IFN-γ levels, with some asthmatics having high IL-13 levels (with and without high IFN-γ), while controls have low IL-13 levels but can still have high IFN-γ levels. Levels of nasosorption IL-13 against IL-5 (*Z*-score normalised) on day 0 were plotted as a 2D cluster, and demonstrate that there is reasonable discrimination between asthmatics (red dots) and healthy volunteers (blue dots) at baseline before infection (Fig. 4C). These features are also present during asthma exacerbation on day 4 (Fig. 4D), when the healthy and allergic asthmatic patients form separate clusters with overlap for only a few subjects: with asthmatic patients generally having higher levels of nasal IL-5 and IL-13.

### 3.8 Bronchial Mucosal Responses to Infection

**Fig. 5:**
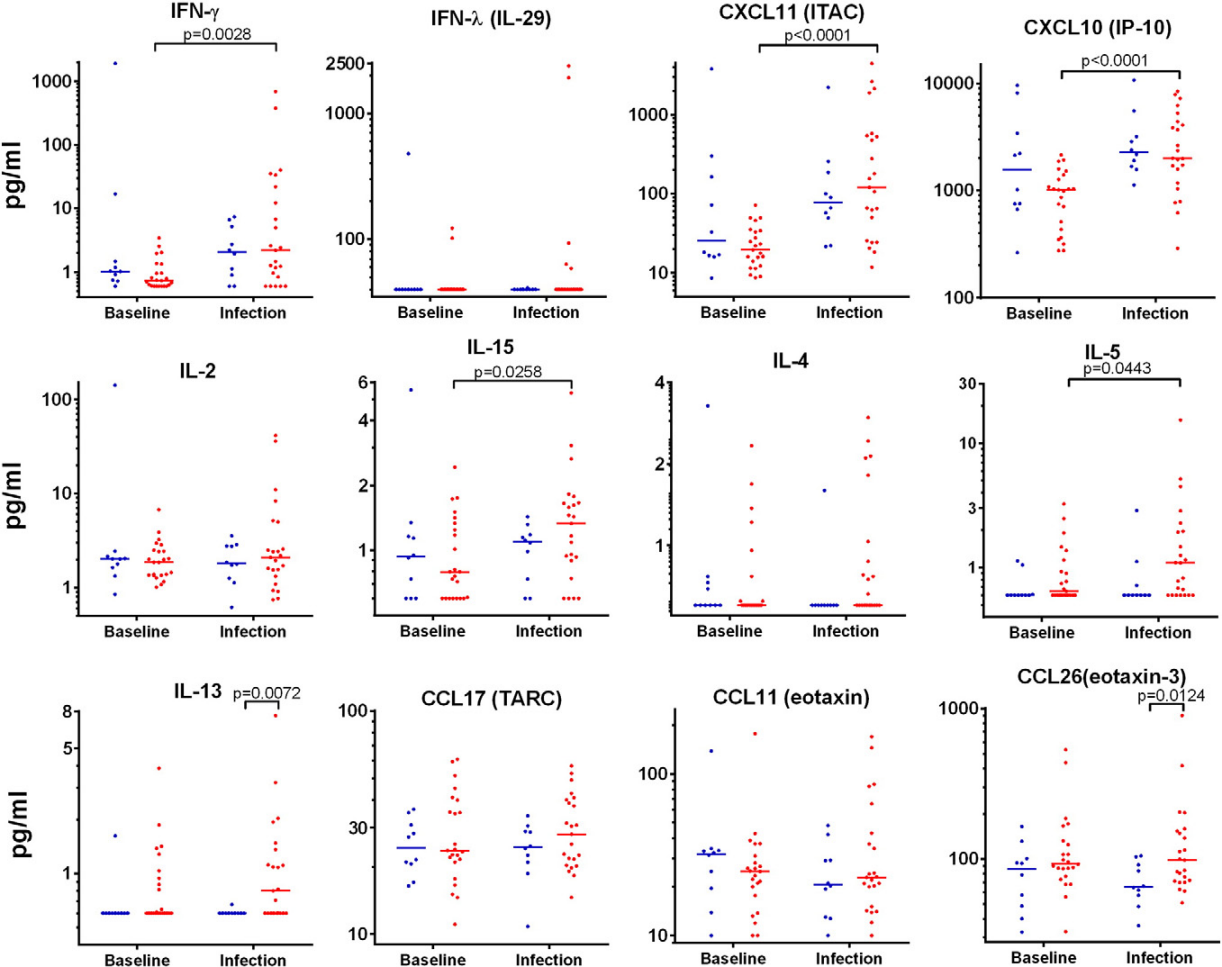
Bronchial mucosal lining fluid levels of 12 cytokines and chemokines. Individual raw data is presented for levels of cytokines and chemokines in bronchial lining fluid, for asthmatic (red) and healthy (blue) subjects. Baseline bronchial samples were taken on day - ~ 14 before inoculation with rhinovirus, while post samples were taken on day 4 post-inoculation. Probability was tested using a Wilcoxon signed rank test: matched/paired samples (baseline/day 4) and the Mann-Whitney test for the unmatched samples (healthy *vs* asthma). Data is shown for asthmatics (*n* = 25) and healthy volunteers (*n* = 11) at baseline and (*n* = 10) for day 4. A full table of summary statistics and probability values is given in the Table S12. For display purposes on a log scale, the lower limit of detection (LLD) was used for all cytokines with values ≤ LLD.

There was significant induction of bronchial IFN-γ, CXCL11/ITAC, CXCL10/IP10, IL-15, IL-10, TNFα and IL-5 during infection in allergic asthmatics (all *P* < 0.05), but not in healthy controls (Fig. 5 and Table S12). We have previously reported that bronchial levels of IL-5 and IL-13 were greater during infection in asthmatic compared with healthy subjects (Jackson et al., 2014).

## 4 Discussion

This study found an amplified immune response to rhinovirus infection in adults with allergic asthma, by using the mucosal sampling methods of nasosorption and bronchosorption to measure a panel of 34 cytokines and chemokines. We observed striking up-regulation of interferon and type 2 inflammatory pathways in our allergic asthmatic subjects. The magnitude of the IFN-γ response to infection is relatively large compared to type 2 cytokine induction, but induction of IL-5 and IL-13 is more selective for allergic asthmatics compared to controls. However, we do not know whether these mediators take part in the pathogenesis of the exacerbation of asthma and/or are part of a protective immune response to combat viral infection. In addition, there was marked heterogeneity between individual asthmatic patients in terms of the relative involvement of these pathways, reflected in mucosal levels of IFN-γ and IFN-λ/IL-29 relative to IL-5 and IL-13. In contrast, in healthy controls there were few significant changes in levels of nasal and bronchial cytokines after rhinovirus infection.

Although there have been previous publications on peak nasal levels of IL-15, IL-18, IL-25, and IL-33 (with IL-5 and IL-13) from this viral challenge study (Jackson et al., 2014, Jackson et al., 2015, Jayaraman et al., 2014); we now provide detailed kinetics of 34 cytokine and chemokine levels in individuals over time. We stress the importance of scrutiny of graphs of data points for individual subjects, comprising cytokine and chemokine levels over time, since a single sample is generally taken from a patient in the context of measuring a biomarker of a disease and making a therapeutic decision. Nasal IL-5 and IL-13 levels were generally < 1 pg/ml in healthy controls, but higher at > 1 pg/ml in most stable allergic asthmatics prior to infection. This demonstrates that for nasal IL-5 and IL-13 levels there is “room to move” in relation to monitoring levels in stable allergic asthmatics. This has major implications for future clinical studies, including dose range finding, of biologics directed against IL-5 or IL-13. Repeat daily nasal sampling could be performed in stable allergic asthmatics in a baseline run-in period, before continued serial nasal sampling after administration of a biologic directed against IL-5 or IL-13. Furthermore, those asthma patients with higher levels of nasal IL-5 and IL-13 prior to RV infection, tended to be those with higher nasal levels of IL-5 and IL-13 after infection. Hence, levels of nasal IL-5 and IL-13 when a patient is stable could be used to select a biologic that acts mainly in the context of exacerbations.

We also found that that cytokines and chemokines of the type 2 pathway were closely related as a group in nasosorption samples in the asthmatic subjects during the 7 days after the infection. This included CCL17/TARC, CCL22/MDC, CCL11/eotaxin and CCL26/eotaxin-3 that are chemokines produced by epithelial and dendritic cells early in the type 2 pathway and are chemotactic for Th2 cells and eosinophils. The relative degree of interferon and type 2 pathway induction was found to vary between asthmatic patients, suggesting that these may be divergent pathways in response to rhinovirus infection. This is in agreement with a recent report that IL-33 promotes, and IFN-γ suppresses, type 2 inflammation (Molofsky et al., 2015), while IFN-λ2 has also been shown to suppress type 2 inflammation (Koltsida et al., 2011).

Nasal levels of IL-5 and IL-13 were tightly correlated with each other in this study and after nasal allergen challenge (Leaker et al., 2017). This suggests that IL-5 and IL-13 could be produced by the same cell type(s), such as by type 2 innate lymphoid cells (ILC2) (Yu et al., 2014, Walker et al., 2013), since ILC2 cells have been demonstrated in the blood and lungs of asthmatics (Barnig et al., 2013, Bartemes et al., 2014). Furthermore, it has recently been shown that IL-25 drives rhinovirus induced allergic inflammation (Beale et al., 2014), and that epithelial-derived IL-33 activates human ILC2 cells and T cells to secrete large amounts of IL-5 and IL-13 (Jackson et al., 2014).

Prior to infection nasal IFN-gamma levels were generally < 2 pg/ml, before rapidly increasing after RV infection, particularly in subjects with allergic asthma. Our finding of elevated nasal and bronchial levels of IFN-γ and IFN-λ (together with the IFN-inducible proteins CXCL10/IP10, CXCL11/ITAC and IL-15) is in agreement with elevated IFN-γ and IFN-λ responses to virus infection being found *in vivo* in children with asthma (Lewis et al., 2012, Miller et al., 2012). However, interferon and IL-15 deficiency have been demonstrated in cultured cells retrieved from stable asthmatics at baseline following infection of these cells with a standardised quantity of rhinovirus *ex vivo* (Wark et al., 2005, Contoli et al., 2006, Edwards et al., 2013, Message et al., 2008, Sykes et al., 2012, Laza-Stanca et al., 2011). Measurement of cytokines and chemokines in nasal MLF was studied in the context of allergic asthmatics challenged with RV. Following experimental nasal allergen challenge (NAC) with grass pollen in allergic asthma and hay fever, we see increases in mediators of type II inflammation in most subjects, but we have not seen an increase in IFN-gamma or related mediators. However, it will also be relevant to measure nasal and bronchial mediators in natural allergen-induced and virally-induced exacerbations.

It would be of interest to compare nasal and bronchial mucosal viral load in allergic asthmatics and healthy controls following RV infection. However, a weakness of the current study was that virus load was measured in nasal lavage samples, when the lavage causes dilution of nasal secretions in 5 ml of saline, and there is variable recovery of fluid. These problems of dilution and variable recovery are also relevant for bronchoalveolar lavage (BAL) samples: where viral load cannot usually be reliably measured. In the future it will be of interest to take nasosorption and bronchosoprtion samples and perform elution in RNA extraction buffer, prior to measuring RV load by qPCR. We observed significantly greater nasal lavage virus load on day 3 but not on day 4 *in vivo* in asthmatic subjects (Jackson et al., 2014). Thus it is possible that the amplified cytokine responses observed *in vivo* in asthmatics may be a consequence of greater virus load. In addition, we did not assess *ex vivo* interferon responses in our subjects at baseline, so we were not able to relate *ex vivo* interferon responses at baseline with *in vivo* virus load during infection.

The technique of nasosorption involves pressing against the mucosa without friction against the surface, as opposed to conventional swab sampling where rotation against the mucosa is required. This has the major advantage of allowing repeat or serial nasosorption sampling at regular intervals. Our group has shown that nasosorption can be repeated every 10 min over an hour, and produce reproducible cytokine and chemokine levels (data not shown).

Nasosorption and bronchosorption of MLF involves sampling from a distinct mucosal compartment, and it is speculated that the cytokines and chemokines detected in MLF reflects the underlying mucosal tissue inflammation. In addition, MLF from the larger bronchi is expected to be influenced by events in the peripheral small airways through the mucociliary escalator. The biological significance of these MLF chemokines and cytokines is open to conjecture, with many chemokines (including CXCL8/IL-8) generally present in respiratory MLF at high levels, while all nasal and bronchial MLF mediators measured have been diluted during elution in immunoassay buffer.

We studied adults with allergic asthma and compared them with healthy non-allergic controls. In exacerbations of asthma taking place in a natural clinical context there may be different signatures of inflammation according to the individual patient's asthma phenotype. It will be relevant to employ nasosorption in large scale natural history and epidemiology studies on a range of asthma phenotypes, including non-allergic and severe asthma. There is also the need to compare nasosorption and bronchosorption sampling techniques with other sampling methods in a range of asthma phenotypes, and to correlate biomarkers with a range of clinical outcomes. Indeed it will be relevant to assess mucosal lining fluid from different locations in the airways in a range of respiratory diseases, to assess whether disease-specific signatures and useful biomarkers can be identified.

This study did not include any therapeutic intervention, but since absorption can be used to quantitate cytokines of the type 2 pathway in the upper and lower airway, future clinical studies could be designed to assess whether these biomarkers can be used to select patients for monoclonal antibody therapy directed against IL-5 and IL-13 (Chung, 2015). Indeed, the need for new biomarkers of inflammation in asthma has been highlighted in a recent study of an IL-13 neutralising monoclonal antibody (Brightling et al., 2015), where measurement of airway IL-13 levels might be a more relevant biomarker than blood eosinophils and serum periostin. Since endobronchial mucosal biopsy in severe asthma is sometimes clinically indicated (Doberer et al., 2015), bronchosorption could be performed during the same bronchoscopy procedure. Hence, we speculate that precision sampling of airway lining fluid, enabling assessment of interferon and type 2 immune responses in the nose and bronchi, has the potential for selection and monitoring of asthmatics in relation to monoclonal antibody therapy.

## Funding Sources

This study was supported by the European Research Council (ERC FP7 grant 233015), a Chair from Asthma UK (CH11SJ), MRC Centre grant G1000758, National Institute of Healthcare Research Biomedical Research Centre (NIHR BRC) grant P26095, Predicta FP7 Collaborative Project grant 260895 by NIHR BRCs at Imperial College London and King’s College London. Novartis Institute for Biomedical Research (Horsham, UK) funded the cytokine assays and development of bronchosorption by Hunt Developments (UK) Ltd, Midhurst, UK. SLJ is an NIHR Senior Investigator. The Funders did not have a role in study design, data collection, data analysis, interpretation, and writing of the report.

## Conflicts of Interest

DJJ has received support for travel expenses to attend Respiratory Conferences from AstraZeneca, Boehringer Ingelheim (UK), and GSK.

SLJ reports grants and/or personal fees from Centocor; Sanofi Pasteur; GSK; Chiesi; Boehringer Ingelheim; Novartis; grants, personal fees and shareholding from Synairgen; personal fees from Bioforce outside the submitted work; In addition, SLJ is involved in patents relating to use of interferon-beta and interferon-lambda for the treatment and prevention of virally-induced exacerbation in asthma and chronic pulmonary obstructive disease, and for induction of cross-reactive cellular responses against rhinovirus antigens.

TTH has been Principal Investigator for respiratory clinical studies carried out at Imperial College with Institutional support from Pharmaceutical Companies: Merck, Novartis, GlaxoSmithKline (GSK), Dainippon-Sumitomo (Sunovion). TTH has received educational grants to attend Respiratory Conferences from Boehringer Ingelheim (UK), and performed occasional consultancy work for Bayer and Retroscreen. TTH together with TMH, TLH, DGH and Imperial Innovations are involved in setting up a medical device company called Mucosal Diagnostics (MD),that is an Imperial College spin-off company.

## Author Contributions

S.L.J., P.M., D.J.J., T.T.H. conceptualized the study design and directed the project; M.-B.T.-T, J.d.-R., and J.D. carried out volunteer screenings and assisted with bronchsoscopies; D.J.J. carried out the clinical aspects of the study; D.J.J. and O.M.K. performed the bronchoscopies; A.T., J.A., L.G., E.B., L.S., B.S., M.J.E., R.P.W. and J.W. performed sample processing and analysis; T.T.H., T.M.H., T.L.H. and D.G.H. designed and produced of nasosorption and bronchosorption devices; T.T.H., T.T., P.K., M.S., J.K., D.J.J. analysed, reviewed and interpreted data; T.T.H., T.T. prepared the manuscript with input from all authors.

## Appendix A Supplementary data

Supplementary material. Image 3
