# Supplementary material for: A Comprehensive Evaluation of Nasal and Bronchial Cytokines and Chemokines Following Experimental Rhinovirus Infection in Allergic Asthma: Increased Interferons (IFN-γ and IFN-λ) and Type 2 Inflammation (IL-5 and IL-13)
Source: eBioMedicine. 2017 Mar 28;19:128–38. doi: 10.1016/j.ebiom.2017.03.033 (PMC5440599; doi:10.1016/j.ebiom.2017.03.033)
Supplement: Supplementary file 1 — Supplementary material. [file mmc1.docx]

**Online Data Supplement:**

**A Comprehensive Evaluation of Nasal and Bronchial Cytokines and Chemokines Following Experimental Rhinovirus Infection in Allergic Asthma: Increased Interferons (IFN-γ and IFN-λ) and Type 2 Inflammation (IL-5 and IL-13)**

Trevor T. Hansel, MD, PhD^1,2,3,4 Ϯ^, Tanushree Tunstall, MSc^3,4^, Maria-Belen Trujillo-Torralbo, MSc^1,2,3^, Betty Shamji, MSc^5^, Ajerico del-Rosario, BSc^1,2,3^, Jaideep Dhariwal, MD, PhD^1,2,3^, Paul D.W. Kirk, PhD^6^, Michael PH Stumpf, PhD^7^, Jens Koopmann, PhD^1,8^, Aurica Telcian, MD, PhD^1,2^, Julia Aniscenko, BSc^1,2^, Leila Gogsadze, BSc^1,2^, Eteri Bakhsoliani, MSc^1,2^, Luminita Stanciu MD, PhD^1,2^, Nathan Bartlett, PhD^1,2^, Michael Edwards, PhD^1,2^, Ross Walton, PhD^1,2^, Patrick Mallia MD, PhD^1,2,3^, Toby M. Hunt^9^, Trevor L. Hunt^9^, Duncan G. Hunt^9^, John Westwick, PhD^5^, Matthew Edwards, PhD^5^, Onn Min Kon, MD, PhD^3,4^, David J. Jackson, MD, PhD^1,2,3,8*^, and Sebastian L. Johnston, MD, PhD^1,2,3*^

From ^1^Airway Disease Infection Section, National Heart and Lung Institute (NHLI), Imperial College (IC), London, UK;

^2^MRC & Asthma UK Centre in Allergic Mechanisms of Asthma;

^3^Imperial College Healthcare NHS Trust;

^4^Imperial Clinical Respiratory Research Unit (ICRRU);

^5^Novartis Institute for Biomedical Research, Horsham, UK;

^6^MRC Biostatistics Unit, Cambridge Institute of Public Health, Cambridge, UK;

^7^Dept. of Theoretical Systems Biology at IC;

^8^Guy’s and St Thomas’ NHS Trust

Contents

[Figure S1. Human Rhinovirus Viral Load Measured by Quantitative Polymerase Chain Reaction (qPCR) in Nasal Lavage Fluid. 3](#_Toc476043636)

[Figure S2. Nasal Mucosal Lining Fluid (MLF) Cytokine and Chemokine Responses (22) to Human Rhinovirus Infection: Medians with Quartiles. 4](#_Toc476043637)

[Figure S3. Nasal Mucosal Lining Fluid (MLF) Cytokine and Chemokine Responses (12) to Human Rhinovirus Infection: Individual data points on a Linear Scale 5](#_Toc476043638)

[Figure S4. Nasal Mucosal Lining Fluid (MLF) Cytokine and Chemokine Responses (22) to Human Rhinovirus Infection: Individual Data Points on a Log Scale. 6](#_Toc476043639)

[Figure S5. Nasal Mucosal Lining Fluid (MLF) Cytokine and Chemokine Responses (22) to Human Rhinovirus Infection: Individual Data Points on a Linear Scale. 7](#_Toc476043640)

[Figure S6. Receiver Operating Characteristic (ROC) Curves for Nasal IL-13 on days 0 & 4 8](#_Toc476043641)

[Table S1 . Correlation of Cytokine and Chemokine Levels in Nasosorption MLF with Viral Load Measured in Nasal Lavage in Allergic Asthmatics (AA) and Healthy Controls (HC) on Day 3 9](#_Toc476043642)

[Table S2. Correlation of Cytokine and Chemokine Levels in Nasosorption MLF with Viral Load Measured in Nasal Lavage in Allergic Asthmatics (AA) and Healthy Controls (HC) on Day 4 10](#_Toc476043643)

[Table S3. Correlation of Cytokine and Chemokine Levels in Nasosorption MLF with Viral Load Measured in Nasal Lavage in Allergic Asthmatics (AA) and Healthy Controls (HC) (AUC Days 2-7) 11](#_Toc476043644)

[Table S4. Comparison of Nasal Mucosal Lining Fluid (MLF) Cytokine and Chemokine 12](#_Toc476043645)

[Levels in Allergic Asthmatics vs Healthy Controls, Area Under Curve (AUC) days 0- 7 12](#_Toc476043646)

[Table S5. Comparison of Nasal MLF Cytokine and Chemokine Levels in Allergic Asthmatics (AA) vs Healthy Controls (HC) on Day 0 and Day 2 13](#_Toc476043647)

[Table S6. Comparison of Nasal MLF Cytokine and Chemokine Levels in Allergic Asthmatics (AA) vs Healthy Controls (HC) on Day 0 and Day 3 14](#_Toc476043648)

[Table S7. Comparison of Nasal MLF Cytokine and Chemokine Levels in Allergic Asthmatics (AA) vs Healthy Controls (HC) on Day 0 and Day 4 15](#_Toc476043649)

[Table S8. Comparison of Nasal MLF Cytokine and Chemokine Levels in Allergic Asthmatics (AA) vs Healthy Controls (HC) on Day 0 and Day 5 16](#_Toc476043650)

[Table S9. Comparison of Nasal MLF Cytokine and Chemokine Levels in Allergic Asthmatics (AA) vs Healthy Controls (HC) on Day 0 and Day 7 17](#_Toc476043651)

[Table S10. Nasosorption Changes in Cytokines and Chemokines from Baseline for Allergic Asthmatics (AA) and Healthy Controls (HC) 18](#_Toc476043652)

[Table S11. Receiver Operating Characteristics (ROC) for Nasal Cytokines and Chemokines on Day 0 and Day 4 in Relation to Allergic Asthmatics (AA) and Healthy Controls (HC) 21](#_Toc476043653)

[Table S12. Comparison of Bronchial MLF Cytokine and Chemokine Levels in Allergic Asthmatics (AA) vs Healthy Controls (HC) at Baseline (BL) and Day 4 22](#_Toc476043654)


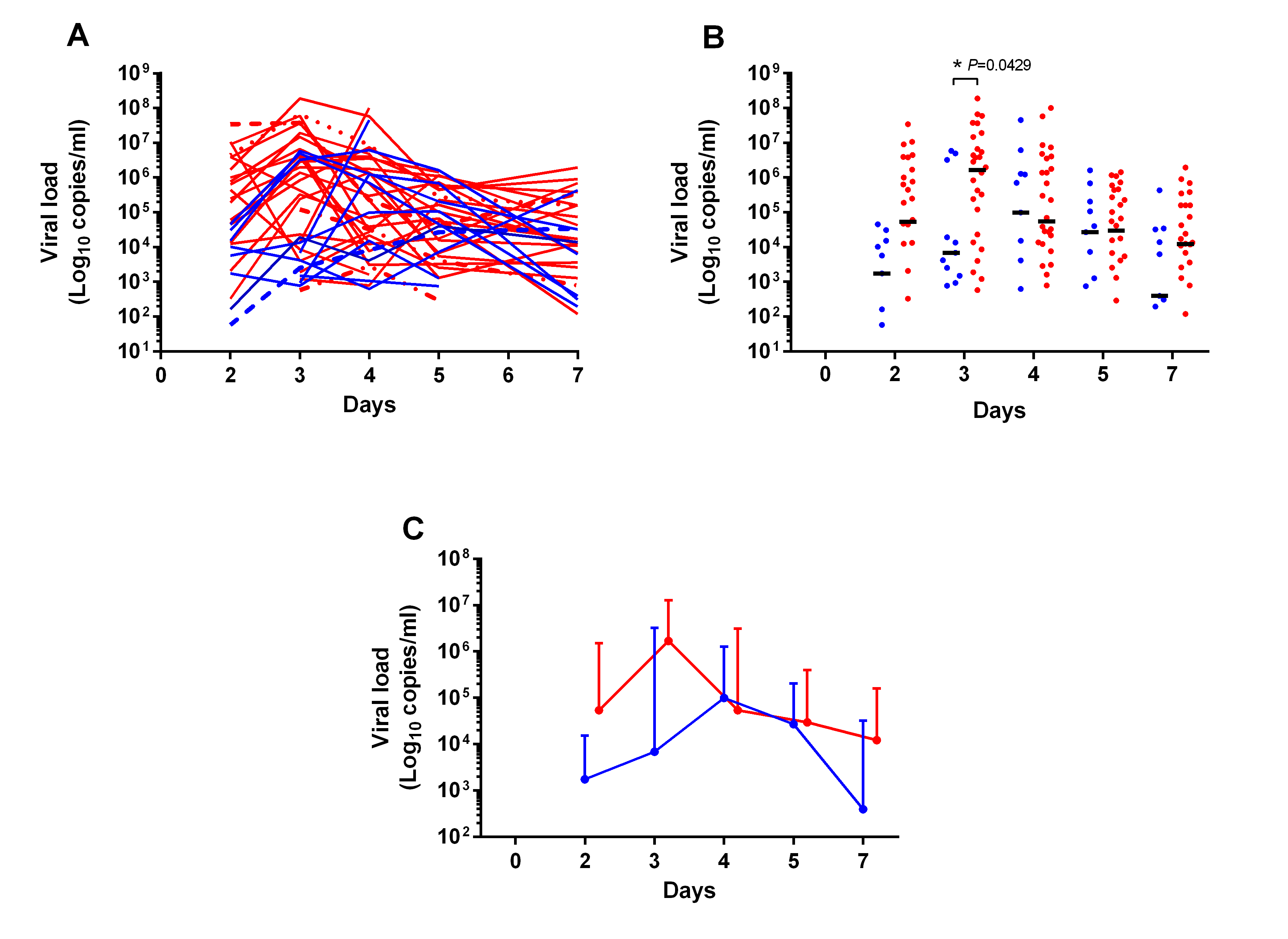


Figure S1. Human Rhinovirus Viral Load Measured by Quantitative Polymerase Chain Reaction (qPCR) in Nasal Lavage Fluid.

1.
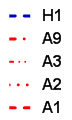
Individual data points are shown for allergic asthmatics (red, n=28) and healthy control subjects (blue, n=11). A single healthy control and 4 asthmatics are given Identifiers (see key, right)
2. Individual data points with medians.
3. Medians and quartiles

Healthy controls and asthmatics had no significant difference in viral load levels (Area Under Curve (AUC) days 2-7), Mann Whitney test, ns, *P*=0.0833.

*Levels of viral load were significantly higher in asthmatics compared to healthy controls on day 3 (Mann Whitney test for unpaired data). Viral load data from this study has been previously published in a different format (1).

1. Jackson DJ, Makrinioti H, Rana BM, Shamji BW, Trujillo-Torralbo MB, Footitt J, Jerico D, Telcian AG, Nikonova A, Zhu J, Aniscenko J, Gogsadze L, Bakhsoliani E, Traub S, Dhariwal J, Porter J, Hunt D, Hunt T, Hunt T, Stanciu LA, Khaitov M, Bartlett NW, Edwards MR, Kon OM, Mallia P, Papadopoulos NG, Akdis CA, Westwick J, Edwards MJ, Cousins DJ, Walton RP, Johnston SL. IL-33-Dependent Type 2 Inflammation during Rhinovirus-induced Asthma Exacerbations In Vivo. *Am J Respir Crit Care Med* 2014; 190: 1373-1382.

**
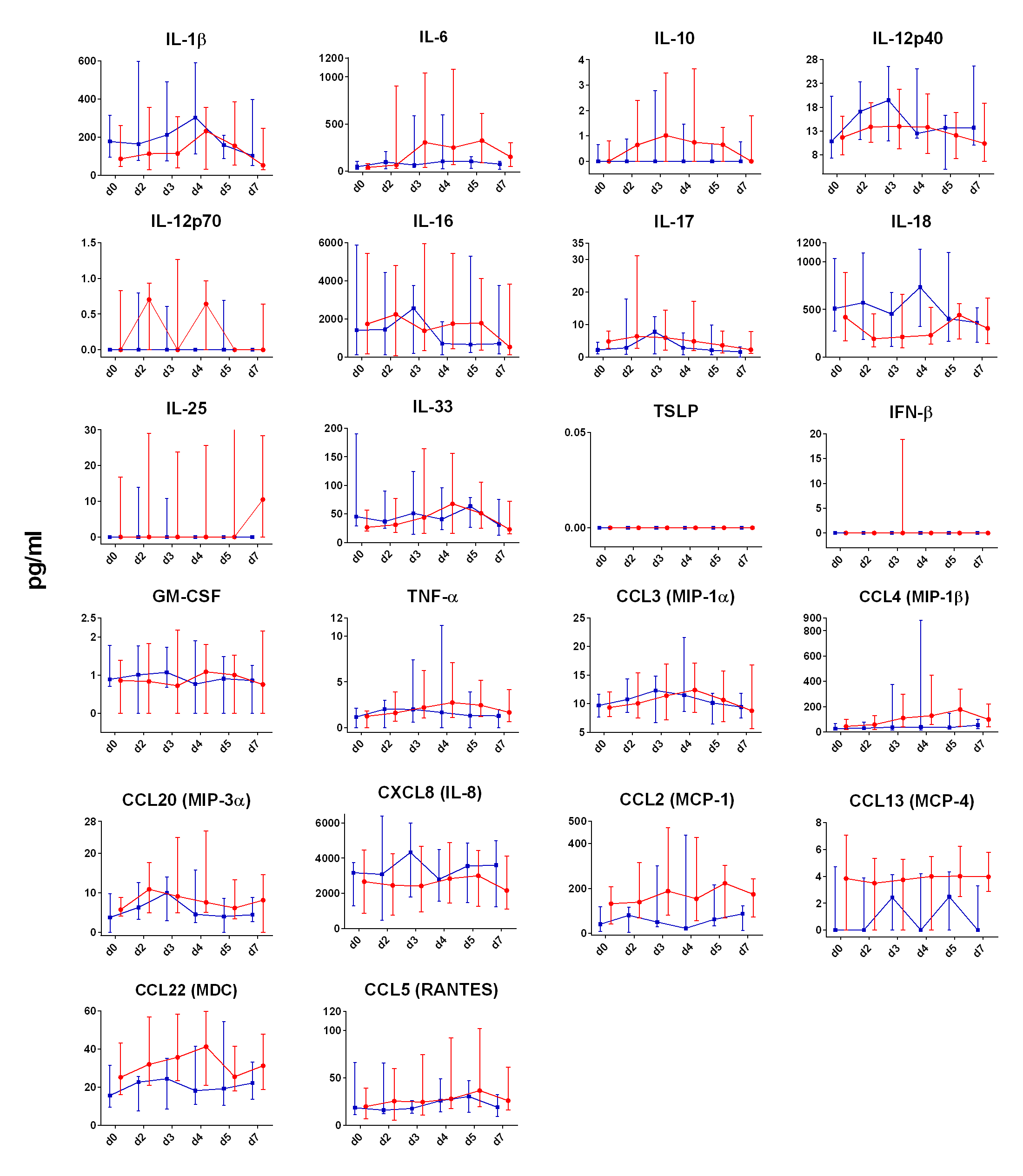
**

Figure S2. Nasal Mucosal Lining Fluid (MLF) Cytokine and Chemokine Responses (22) to Human Rhinovirus Infection: Medians with Quartiles. Median levels with quartiles for allergic asthmatics (red, n=28) and healthy control subjects (blue, n=11). Levels of 22 cytokines and chemokines in nasal MLF obtained by nasosorption were determined by multiplex immunoassay. d= days

**

**

Figure S3. Nasal Mucosal Lining Fluid (MLF) Cytokine and Chemokine Responses (12) to Human Rhinovirus Infection: Individual data points on a Linear Scale. Levels of 12 cytokines and chemokines in nasal mucosal lining fluid (MLF) obtained by nasosorption were determined by multiplex immunoassay. Individual values for all subjects: allergic asthmatics (red, n=28) and healthy control subjects (blue, n=11). Four representative asthmatics and one healthy volunteer are given specific line patterns to allow identification of these individuals across the panel of cytokine responses. These cytokines and chemokines have been presented on a log scale in Fig.2 of the manuscript. d=days


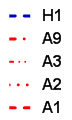


**

**

Figure S4. Nasal Mucosal Lining Fluid (MLF) Cytokine and Chemokine Responses (22) to Human Rhinovirus Infection: Individual Data Points on a Log Scale. Levels of 22 cytokines and chemokines in nasal MLF obtained by nasosorption were determined by multiplex immunoassay. Four representative asthmatics and one healthy volunteer are given specific line patterns to allow identification of these individuals across the panel of cytokine responses. d=days.


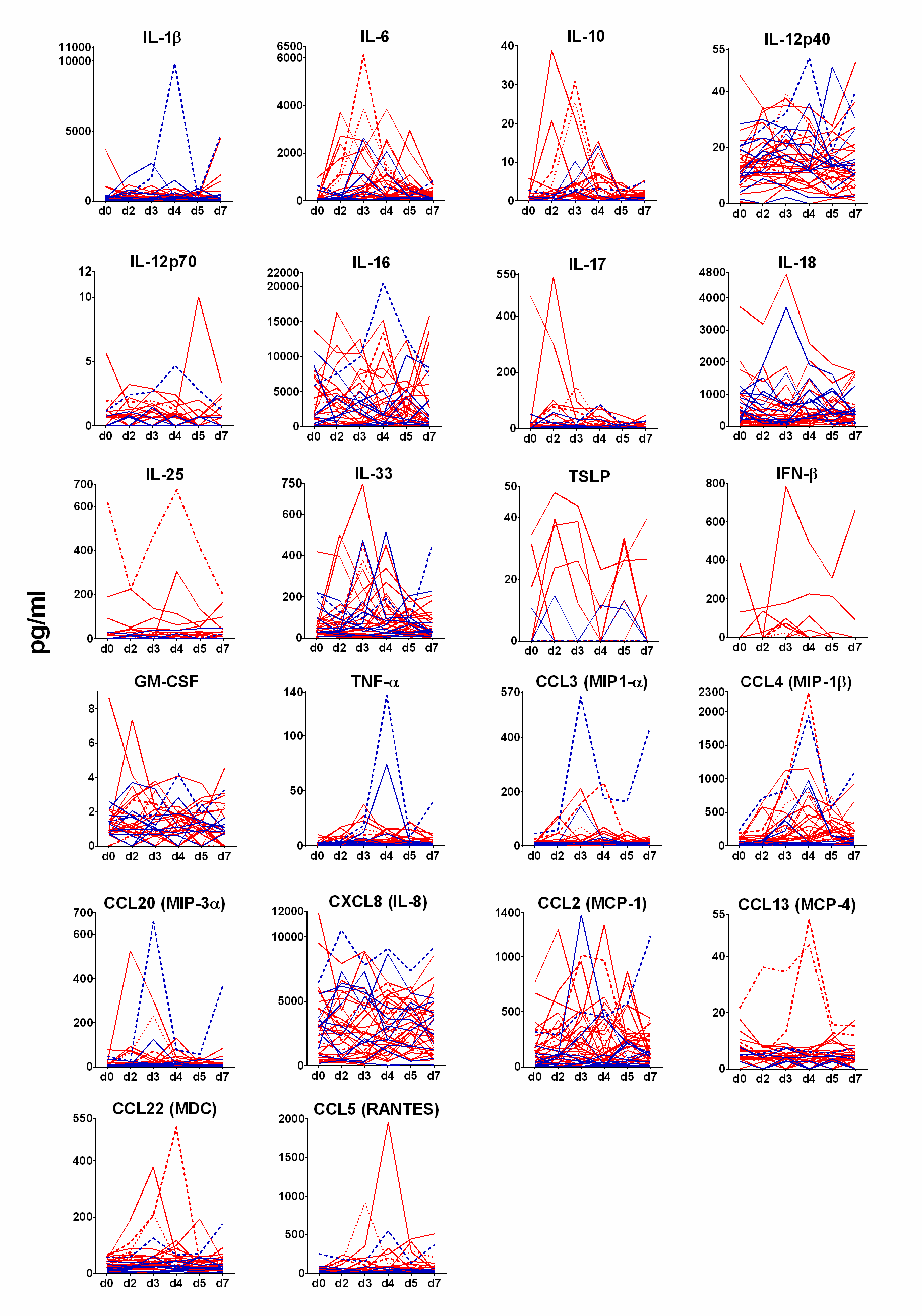

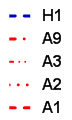


Figure S5. Nasal Mucosal Lining Fluid (MLF) Cytokine and Chemokine Responses (22) to Human Rhinovirus Infection: Individual Data Points on a Linear Scale. Levels of 22 cytokines and chemokines in nasal MLF obtained by nasosorption were determined by multiplex immunoassay. Four representative asthmatics and one healthy volunteer are given specific line patterns to allow identification of these individuals across the panel of cytokine responses.





##
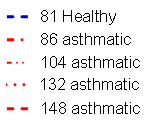
Figure S6. Receiver Operating Characteristic (ROC) Curves for Nasal IL-13 on days 0 & 4

The Receiver Operating Characteristic (ROC) curve analyses were performed on log normalised data using the Monte Carlo cross-validation method, and used a predictive model based on linear discriminate analysis (LDA).

The red lines show that IL-13 levels can classify asthma patients from healthy controls.

The black lines (permuted data) show the prediction accuracy for the study data when the asthma and control status was randomized. This shows that the feature selection and classification algorithm is unbiased and does not overestimate the prediction accuracy.

The prediction accuracy was calculated using nested cross-validation and it achieves an area under the curve (AUC) on day 0=0.75 and on day 4=0.84.

The optimum performance point (OPP) is indicated by the small blue circle, and is that point on the ROC curve with a maximal sum of sensitivity and specificity.

## Table S1 . Correlation of Cytokine and Chemokine Levels in Nasosorption MLF with Viral Load Measured in Nasal Lavage in Allergic Asthmatics (AA) and Healthy Controls (HC) on Day 3

| **Cytokine**  **Chemokine** | **Day 3 VL**  **Day 3 HC**  **Correlation**  ***Spearman rho(r_s_)*** | **Day 3 VL**  **Day 3 AA**  **Correlation**  ***Spearman rho(r_s_)*** |
| --- | --- | --- |
| **IL-1β** | 0.1455 | 0.2381 |
| **IL-2** | -0.0727 | 0.2222 |
| **IL-4** | -0.2081 | -0.0284 |
| **IL-5** | -0.1369 | 0.0304 |
| **IL-6** | -0.1182 | 0.3098 |
| **IL-10** | -0.2211 | 0.3427 |
| **IL-12p40** | -0.3818 | 0.1916 |
| **IL-12p70** | -0.6244 (P= 0.0424) * | 0.2373 |
| **IL-13** | -0.4106 | -0.0944 |
| **IL-15** | -0.1273 | 0.4530 (*P*=0.0155) * |
| **IL-16** | -0.2091 | 0.2551 |
| **IL-17** | -0.0182 | 0.2770 |
| **IL-18** | 0.4636 | -0.2239 |
| **IL-25** | 0.4336 | 0.0010 |
| **IL-33** | -0.0455 | 0.4953 (*P*=0.0074) ** |
| **IFN-γ** | -0.2956 | 0.3190 |
| **IFN-β** | NA | 0.3368 |
| **IFN-λ (IL-29)** | -0.4683 | 0.1971 |
| **GM-CSF** | -0.1868 | 0.2009 |
| **TNF-α** | -0.2961 | 0.3449 |
| **TSLP** | NA | -0.0234 |
| **CCL2/MCP-1** | -0.3364 | 0.3903 (*P*=0.0401) * |
| **CCL3/MIP-1α** | -0.2000 | 0.4439 (*P*=0.0180) * |
| **CCL4/MIP-1β** | -0.0364 | 0.3612 |
| **CCL5/RANTES** | -0.3727 | 0.3043 |
| **CCL11/eotaxin** | -0.4100 | 0.2828 |
| **CCL13/MCP-4** | -0.4005 | 0.1920 |
| **CCL17 /TARC** | -0.3455 | 0.0564 |
| **CCL20/MIP-3α** | -0.3371 | 0.3408 |
| **CCL22 /MDC** | -0.4636 | 0.2737 |
| **CCL26/eotaxin-3** | -0.2273 | 0.0104 |
| **CXCL8/IL-8** | -0.0636 | 0.3744 (*P*=0.0497) * |
| **CXCL10/ IP10** | -0.3455 | 0.2408 |
| **CXCL11 /ITAC** | -0.3182 | 0.3207 |

HC=healthy controls, AA=allergic asthmatics

Levels of nasal cytokines and chemokines were correlated with viral load 3 in HC (n=11) and in AA (n=28) on day 3

Samples with values under lower limit of detection (LLD) were given a value of 0 pg/ml.

## Table S2. Correlation of Cytokine and Chemokine Levels in Nasosorption MLF with Viral Load Measured in Nasal Lavage in Allergic Asthmatics (AA) and Healthy Controls (HC) on Day 4

| **Cytokine**  **Chemokine** | **Day 4 VL**  **Day 4 HC**  **Correlation**  ***Spearman rho(r_s_)*** | **Day 4 VL**  **Day 4 AA**  **Correlation**  ***Spearman rho(r_s_)*** |
| --- | --- | --- |
| **IL-1β** | -0.0319 | 0.0542 |
| **IL-2** | -0.1549 | -0.0036 |
| **IL-4** | -0.3041 | 0.0046 |
| **IL-5** | -0.1689 | -0.1183 |
| **IL-6** | -0.0137 | -0.0104 |
| **IL-10** | -0.2636 | 0.0339 |
| **IL-12p40** | -0.5103 | 0.1574 |
| **IL-12p70** | -0.2973 | 0.0485 |
| **IL-13** | -0.5752 | 0.0118 |
| **IL-15** | -0.0046 | 0.0386 |
| **IL-16** | -0.2460 | 0.1092 |
| **IL-17** | -0.3288 | 0.0213 |
| **IL-18** | 0.3690 | -0.1426 |
| **IL-25** | -0.0676 | 0.0963 |
| **IL-33** | -0.0592 | 0.0342 |
| **IFN-γ** | -0.1793 | 0.0318 |
| **IFN-β** | NA | 0.4240 (*P*=0.0245*) |
| **IFN-λ (IL-29)** | -0.3007 | 0.1538 |
| **GM-CSF** | -0.2378 | -0.0097 |
| **TNF-α** | -0.3264 | 0.1724 |
| **TSLP** | 0.2005 | -0.1707 |
| **CCL2/MCP-1** | -0.1276 | 0.2611 |
| **CCL3/MIP-1α** | -0.3690 | 0.2119 |
| **CCL4/MIP-1β** | 0.0457 | 0.1787 |
| **CCL5/RANTES** | -0.3781 | 0.0859 |
| **CCL11/eotaxin** | -0.2752 | 0.1442 |
| **CCL13/MCP-4** | -0.5277 | 0.3744 (*P=*0.0497*) |
| **CCL17 /TARC** | -0.4146 | 0.2735 |
| **CCL20/MIP-3α** | -0.4749 | 0.2231 |
| **CCL22 /MDC** | -0.4374 | 0.3490 |
| **CCL26/eotaxin-3** | -0.4419 | 0.2264 |
| **CXCL8/IL-8** | -0.3098 | 0.2058 |
| **CXCL10/ IP10** | -0.1868 | 0.1292 |
| **CXCL11 /ITAC** | -0.1412 | 0.0375 |

HC=healthy controls, AA=allergic asthmatics

Levels of nasal cytokines and chemokines were correlated with viral load in HC (n=11) and in AA on day 4 (n=28).

Samples with values under lower limit of detection (LLD) were given a value of 0 pg/ml.

All significant correlations are highlighted in pink, and corresponding P-values are shown.

## Table S3. Correlation of Cytokine and Chemokine Levels in Nasosorption MLF with Viral Load Measured in Nasal Lavage in Allergic Asthmatics (AA) and Healthy Controls (HC) (AUC Days 2-7)

| **Cytokine**  **Chemokine** | **AUC VL**  **AUC HC**  **Correlation**  ***Spearman rho(r_s_)*** | **AUC VL**  **AUC AA**  **Correlation**  ***Spearman rho(r_s)_*** |
| --- | --- | --- |
| **IL-1β** | 0.1545 | 0.1505 |
| **IL-2** | -0.0182 | 0.2731 |
| **IL-4** | -0.2422 | -0.0723 |
| **IL-5** | 0.0095 | -0.1757 |
| **IL-6** | -0.1545 | 0.2556 |
| **IL-10** | -0.2094 | 0.2103 |
| **IL-12p40** | -0.3364 | 0.1959 |
| **IL-12p70** | -0.553 | 0.2355 |
| **IL-13** | -0.1284 | -0.0843 |
| **IL-15** | -0.2727 | 0.2261 |
| **IL-16** | -0.2545 | 0.0701 |
| **IL-17** | -0.1273 | 0.2512 |
| **IL-18** | 0.5182 | -0.0148 |
| **IL-25** | -0.2574 | -0.0147 |
| **IL-33** | -0.1455 | 0.3859* (P=0.0425) |
| **IFN-γ** | -0.3364 | 0.2162 |
| **IFN-β** | NA | 0.5038** (P=0063) |
| **IFN-λ (IL-29)** | -0.461 | 0.2788 |
| **GM-CSF** | -0.1182 | 0.0856 |
| **TNF-α** | -0.2545 | 0.4319* (P=0.0217) |
| **TSLP** | 0.3584 | -0.1414 |
| **CCL2/MCP-1** | -0.4182 | 0.3974* (P=0.0363) |
| **CCL3/MIP-1α** | -0.3545 | 0.3454 |
| **CCL4/MIP-1β** | 0.0182 | 0.4275* (P=0.0233) |
| **CCL5/RANTES** | -0.4091 | 0.2135 |
| **CCL11/eotaxin** | -0.7246* (*P*=0.0162) | 0.1793 |
| **CCL13/MCP-4** | -0.2569 | 0.1664 |
| **CCL17 /TARC** | -0.5545 | 0.0909 |
| **CCL20/MIP-3α** | -0.5513 | 0.2819 |
| **CCL22 /MDC** | -0.5545 | 0.2961 |
| **CCL26/eotaxin-3** | -0.4909 | 0.0142 |
| **CXCL8/IL-8** | 0.0636 | 0.2523 |
| **CXCL10/ IP10** | -0.3091 | 0.1702 |
| **CXCL11 /ITAC** | -0.2455 | 0.1686 |

HC=healthy controls, AA=allergic asthmatics

AUC of nasal cytokines and chemokines were correlated with AUC viral load in HC (n=11) and in AA (n=28) from day 2 to day 7. Samples with values under lower limit of detection (LLD) were given a value of 0 pg/ml.

All significant correlations are highlighted in pink, and corresponding P-values are shown.

## Table S4. Comparison of Nasal Mucosal Lining Fluid (MLF) Cytokine and Chemokine

## Levels in Allergic Asthmatics vs Healthy Controls, Area Under Curve (AUC) days 0- 7

| **Cytokine or**  **chemokine** | **Healthy controls**  **AUC (days 0-7)**  **Median**  **(CIs)** | **Allergic asthmatics**  **AUC (days 0-7)**  **Median**  **(CIs)** | ***P*-values** |
| --- | --- | --- | --- |
| **IL-1β** | 1565.77 (1006.59, 3629.19) | 1192.94 (522.62, 1729.48) | 0.3464 |
| **IL-2** | 11.74 (7.98, 22.64) | 25.00 (18.53, 33.99) | 0.0095 |
| **IL-4** | 0.00 (0.00, 5.75) | 4.47 (2.29, 6.85) | 0.0163 |
| **IL-5** | 1.61 (0.00, 15.01) | 31.52 (18.39, 53.97) | 0.0003 |
| **IL-6** | 906.35 (277.74, 2860.90) | 2302.66 (1109.15, 3943.23) | 0.1399 |
| **IL-10** | 1.43 (0.00, 11.67) | 8.61 (4.30, 14.66) | 0.0666 |
| **IL-12p40** | 113.18 (68.57, 174.78) | 94.90 (76.73, 110.82) | 0.4138 |
| **IL-12p70** | 0.79 (0.00, 2.10) | 1.98 (1.08, 3.81) | 0.1633 |
| **IL-13** | 1.55 (0.00, 6.00) | 19.23 (12.47, 25.90) | <0.0001 |
| **IL-15** | 9.52 (6.85, 21.00) | 19.05 (13.80, 21.96) | 0.0429 |
| **IL-16** | 12155.70 (2040.64, 23175.60) | 12518.89 (8334.10, 28115.07) | 0.7236 |
| **IL-17** | 26.29 (12.02, 142.66) | 48.14 (24.76, 85.76) | 0.2589 |
| **IL-18** | 4723.01 (2116.22, 5985.12) | 2537.17 (1612.78, 3808.44) | 0.1316 |
| **IL-25** | 10.84 (0.00, 24.24) | 22.15 (0.00, 109.09) | 0.3094 |
| **IL-33** | 427.92 (192.60, 845.06) | 392.69 (310.83, 609.90) | 0.5690 |
| **IFN-γ** | 6.54 (1.43, 121.08) | 48.95 (36.44, 161.21) | 0.0470 |
| **IFN-β** | 0.00 (0.00, 0.00) | 0.00 (0.00, 25.19) | 0.0368 |
| **IFN-λ (IL-29)** | 0.00 (0.00, 182.60) | 339.14 (120.52, 570.73) | 0.0126 |
| **GM-CSF** | 7.43 (2.97, 10.84) | 5.86 (3.73, 10.86) | 0.9938 |
| **TNF-α** | 14.05 (1.68, 24.93) | 20.23 (9.35, 30.12) | 0.4882 |
| **TSLP** | 1. (0.00, 19.74) | 0.00 (0.00, 0.00) | 0.9860 |
| **CCL2/MCP-1** | 426.06 (339.89, 1227.22) | 1467.49 (908.43, 2019.77) | 0.0337 |
| **CCL3/MIP-1α** | 84.03 (54.79, 94.41) | 82.13 (65.22, 118.53) | 0.7468 |
| **CCL4/MIP-1β** | 233.15 (175.65, 1638.49) | 1092.57 (636.39, 1402.14) | 0.1486 |
| **CCL5/RANTES** | 178.40 (89.31, 304.76) | 208.29 (150.77, 412.07) | 0.3963 |
| **CCL11/eotaxin** | 33.30 (0.00, 140.09) | 175.62 (128.33, 245.51) | 0.0124 |
| **CCL13/MCP-4** | 5.15 (0.00, 31.82) | 24.17 (17.26, 33.05) | 0.0170 |
| **CCL17/TARC** | 81.99 (48.48, 105.04) | 153.72 (104.09, 179.81) | 0.0115 |
| **CCL20/MIP-3α** | 45.59 (17.57, 162.22) | 74.04 (50.69, 114.44) | 0.3005 |
| **CCL22/MDC** | 151.85 (77.02, 225.39) | 209.44 (175.28, 346.49) | 0.0582 |
| **CCL26/eotaxin-3** | 769.56 (491.47, 912.66) | 1216.36 (954.25, 1454.40) | 0.0184 |
| **CXCL8/IL-8** | 23334.70 (11251.75, 36486.34) | 18190.50 (13761.01, 26939.27) | 0.5690 |
| **CXCL10/IP10** | 14498.42 (5206.66, 19631.79) | 24761.51 (19664.70, 32225.89) | 0.0127 |
| **CXCL11/ITAC** | 500.88 (250.24, 5663.79) | 5149.40 (2982.16, 7572.08) | 0.0285 |

*P*-values are derived from the Mann Whitney Test (non-paired), in allergic asthmatics (n=28) vs. healthy controls (n=11). Significant *P*-values (<0.05) are shaded in pink. 2 missing values were computed by linear interpolation. Samples with values under the lower limit of detection (LLD) were given a value of 0 pg/ml for the analysis.

## Table S5. Comparison of Nasal MLF Cytokine and Chemokine Levels in Allergic Asthmatics (AA) vs Healthy Controls (HC) on Day 0 and Day 2

| **Cytokine**  **Chemokine** | **HC**  **Day 0**  **Median** | **HC**  **Day 2**  **Median** | **AA**  **Day 0**  **Median** | **AA**  **Day 2**  **Median** | **HC**  **Day 0 vs 2**  ***P*** | **AA**  **Day 0 vs 2**  ***P*** | **HC vs AA**  **Day 0**  ***P*** | **HC vs AA**  **Day 2**  ***P*** |
| --- | --- | --- | --- | --- | --- | --- | --- | --- |
| **IL-1β** | 176.99 | 163.03 | 85.94 | 80.05 | 0.6377 | 0.1019 | 0.2816 | 0.0833 |
| **IL-2** | 1.28 | 1.40 | 1.89 | 3.58 | 0.4648 | 0.0155 | 0.2007 | 0.0301 |
| **IL-4** | 0 | 0 | 0.89 | 0.84 | 1.0000 | 0.7756 | 0.0416 | 0.0906 |
| **IL-5** | 0 | 0 | 1.81 | 1.75 | 1.0000 | 0.1045 | 0.0088 | 0.0036 |
| **IL-6** | 46.96 | 95.91 | 40.07 | 65.41 | 0.7002 | 0.0149 | 04633 | 0.7431 |
| **IL-10** | 0 | 0 | 0 | 0.64 | 0.6250 | 0.0104 | 0.5731 | 0.0564 |
| **IL-12p40** | 10.81 | 17.11 | 11.66 | 13.93 | 0.0322 | 0.0315 | 0.8883 | 0.4170 |
| **IL-12p70** | 0 | 0 | 0 | 0.69 | 0.1250 | 0.3778 | 0.1088 | 0.4016 |
| **IL-13** | 0 | 0.63 | 1.95 | 1.96 | 0.9375 | 0.1978 | 0.0015 | 0.0012 |
| **IL-15** | 0.87 | 1.03 | 1.67 | 1.62 | 0.8457 | 0.4903 | 0.2354 | 0.1046 |
| **IL-16** | 1409.01 | 1440.57 | 1737.30 | 2236.52 | 0.8984 | 0.8419 | 0.6066 | 0.7669 |
| **IL-17** | 2.23 | 2.89 | 4.86 | 6.30 | 0.1748 | 0.0438 | 0.1149 | 0.2679 |
| **IL-18** | 509.79 | 571.56 | 418.36 | 188.83 | 0.7646 | 0.0026 | 0.2546 | 0.0511 |
| **IL-25** | 0 | 0 | 0 | 0 | 0.5000 | 0.2661 | 0.2283 | 0.2771 |
| **IL-33** | 45.46 | 36.76 | 26.52 | 27.89 | 0.0537 | 0.2527 | 0.0590 | 0.6735 |
| **IFN-γ** | 0 | 1.01 | 0 | 1.99 | 0.9375 | 0.0012 | 0.8867 | 0.1613 |
| **IFN-β** | 0 | 0 | 0 | 0 | . | 1.0000 | 0.3913 | 0.2799 |
| **IFN-λ (IL-29)** | 0 | 0 | 0 | 74.42 | 0.5000 | 0.0002 | 0.0656 | 0.0101 |
| **GM-CSF** | 0.89 | 1.01 | 0.86 | 0.83 | 0.9102 | 0.3884 | 0.4013 | 0.8493 |
| **TNF-α** | 1.17 | 2.01 | 1.25 | 1.58 | 0.0742 | 0.0032 | 0.9495 | 0.8629 |
| **TSLP** | 0 | 0 | 0 | 0 | 1.0000 | 0.4375 | 0.8357 | 0.6098 |
| **CCL2/MCP-1** | 40.00 | 79.70 | 131.52 | 150.92 | 0.9658 | 0.0379 | 0.0778 | 0.0237 |
| **CCL3/MIP-1α** | 9.67 | 10.73 | 9.32 | 10.03 | 0.0537 | 0.1459 | 0.8149 | 0.6509 |
| **CCL4/MIP-1β** | 26.21 | 29.17 | 43.35 | 56.07 | 0.2324 | 0.0751 | 0.2958 | 0.5120 |
| **CCL5/RANTES** | 18.55 | 16.00 | 19.90 | 23.77 | 0.5771 | 0.0639 | 0.7669 | 0.8149 |
| **CCL11/eotaxin** | 0 | 0 | 16.13 | 18.00 | 0.4375 | 0.0820 | 0.0265 | 0.0139 |
| **CCL13/MCP-4** | 0 | 0 | 3.84 | 3.40 | 0.3750 | 0.0246 | 0.0559 | 0.0610 |
| **CCL17 /TARC** | 8.18 | 10.76 | 17.08 | 19.59 | 0.0371 | 0.0052 | 0.0096 | 0.0192 |
| **CCL20/MIP-3α** | 3.78 | 6.32 | 5.76 | 10.23 | 0.7344 | <0.0001 | 0.4530 | 0.3489 |
| **CCL22 /MDC** | 15.68 | 22.67 | 25.20 | 30.65 | 0.3652 | 0.0029 | 0.0727 | 0.0590 |
| **CCL26/eotaxin-3** | 121.10 | 100.04 | 153.86 | 157.79 | 0.3652 | 0.1763 | 0.0441 | 0.0256 |
| **CXCL8/IL-8** | 3171.24 | 3077.87 | 2662.59 | 2448.36 | 0.5915 | 0.3252 | 0.6964 | 0.3572 |
| **CXCL10/ IP10** | 945.72 | 1531.22 | 1966.10 | 2880.47 | 0.5771 | 0.0160 | 0.1556 | 0.0727 |
| **CXCL11 /ITAC** | 14.51 | 28.83 | 39.28 | 105.94 | 0.2738 | 0.0132 | 0.0861 | 0.0441 |

HC=healthy controls, AA=allergic asthmatics

Levels of nasal cytokines and chemokines were compared in HC from day 0 to day 2 (n=11) and in AA from day 0 to day 2 (n=28).

Samples with values under lower limit of detection (LLD) were given a value of 0 pg/ml.

Paired samples were analysed by the Wilcoxon signed-rank test.

Levels of nasal cytokines and chemokines were compared on day 2 in AA versus HC, using the Mann-Whitney test.

*P*-values associated with each test are shown, with significant values highlighted in pink/blue.

Note that IL-18 and CCL13/MCP-4 levels were decreased after HRV infection in asthmatics highlighted in blue.

## Table S6. Comparison of Nasal MLF Cytokine and Chemokine Levels in Allergic Asthmatics (AA) vs Healthy Controls (HC) on Day 0 and Day 3

| **Cytokine**  **Chemokine** | **HC**  **Day 0**  **Median** | **HC**  **Day 3**  **Median** | **AA**  **Day 0**  **Median** | **AA**  **Day 3**  **Median** | **HC**  **Day 0 vs 3**  ***P*** | **AA**  **Day 0 vs 3**  ***P*** | **HC vs AA**  **Day 3**  ***P*** |
| --- | --- | --- | --- | --- | --- | --- | --- |
| **IL-1β** | 176.99 | 211.49 | 85.94 | 113.63 | 0.3652 | 0.6896 | 0.3412 |
| **IL-2** | 1.28 | 1.75 | 1.89 | 3.83 | 0.8311 | 0.0002 | 0.0590 |
| **IL-4** | 0 | 0 | 0.89 | 1.05 | 1.0000 | 0.2497 | 0.0328 |
| **IL-5** | 0 | 0 | 1.81 | 4.82 | 0.6875 | 0.0002 | 0.0024 |
| **IL-6** | 46.96 | 66.44 | 40.07 | 304.96 | 0.4648 | <.0001 | 0.4825 |
| **IL-10** | 0 | 0 | 0 | 1.02 | 0.3125 | 0.0082 | 0.4303 |
| **IL-12p40** | 10.80 | 19.46 | 11.66 | 13.96 | 0.0420 | 0.0454 | 0.3572 |
| **IL-12p70** | 0 | 0 | 0 | 0 | 0.5000 | 0.4332 | 0.2858 |
| **IL-13** | 0 | 0 | 1.95 | 2.57 | 0.8438 | 0.0016 | 0.0001 |
| **IL-15** | 0.87 | 1.07 | 1.67 | 2.41 | 0.7646 | 0.0155 | 0.1745 |
| **IL-16** | 1409.01 | 2558.91 | 1737.30 | 1376.45 | 0.3652 | 0.5189 | 1.0000 |
| **IL-17** | 2.23 | 7.81 | 4.86 | 6.00 | 0.2061 | 0.1178 | 0.6735 |
| **IL-18** | 509.79 | 452.76 | 418.36 | 211.59 | 0.4648 | 0.1916 | 0.6066 |
| **IL-25** | 0 | 0 | 0 | 0 | 0.2500 | 0.6772 | 0.4239 |
| **IL-33** | 45.46 | 51.23 | 26.52 | 43.93 | 0.7002 | 0.0357 | 0.9627 |
| **IFN-γ** | 0 | 0.86 | 0 | 4.80 | 0.5781 | <.0001 | 0.1278 |
| **IFN-β** | 0 | 0 | 0 | 0 | . | 0.0156 | 0.0763 |
| **IFN-λ (IL-29)** | 0 | 0 | 0 | 21.25 | 0.5000 | 0.0335 | 0.0948 |
| **GM-CSF** | 0.89 | 1.07 | 0.86 | 0.72 | 0.8457 | 0.4896 | 0.6025 |
| **TNF-α** | 1.17 | 2.00 | 1.25 | 2.21 | 0.1055 | 0.0006 | 0.7430 |
| **TSLP** | 0 | 0 | 0 | 0 | 1.0000 | 0.6250 | 0.2026 |
| **CCL2/MCP-1** | 40.00 | 49.82 | 131.52 | 187.59 | 0.1475 | 0.1618 | 0.2295 |
| **CCL3/MIP-1α** | 9.67 | 12.30 | 9.32 | 11.39 | 0.1475 | 0.0276 | 0.7669 |
| **CCL4/MIP-1β** | 26.21 | 37.44 | 43.35 | 109.90 | 0.1475 | <.0001 | 0.2356 |
| **CCL5/RANTES** | 18.55 | 17.82 | 19.90 | 24.58 | 0.4131 | 0.0335 | 0.4445 |
| **CCL11/eotaxin** | 0 | 10.28 | 16.13 | 23.42 | 0.1563 | 0.0396 | 0.0575 |
| **CCL13/MCP-4** | 0 | 2.43 | 3.84 | 3.74 | 0.3125 | 0.3885 | 0.2500 |
| **CCL17/TARC** | 8.18 | 14.64 | 17.08 | 22.60 | 0.0322 | 0.0016 | 0.0301 |
| **CCL20/MIP-3α** | 3.78 | 9.96 | 5.76 | 9.09 | 0.1289 | 0.0024 | 0.8392 |
| **CCL22/MDC** | 15.68 | 24.45 | 25.20 | 35.72 | 0.0674 | 0.0003 | 0.1556 |
| **CCL26/eotaxin-3** | 121.10 | 106.81 | 153.86 | 146.14 | 0.8984 | 0.2623 | 0.0301 |
| **CXCL8/IL-8** | 3171.24 | 4323.79 | 2662.59 | 2419.64 | 0.2402 | 0.2341 | 0.3104 |
| **CXCL10/IP10** | 945.72 | 2256.63 | 1966.10 | 3660.60 | 0.3203 | 0.0010 | 0.0475 |
| **CXCL11/ITAC** | 14.51 | 63.72 | 39.28 | 596.96 | 0.1748 | 0.0001 | 0.0679 |

HC=healthy controls, AA=allergic asthmatics

Levels of nasal cytokines and chemokines were compared in HC from day 0 to day 3 (n=11) and in AA from day 0 to day 3 (n=28).

Samples with values under lower limit of detection (LLD) were given a value of 0 pg/ml.

Paired samples were analysed by the Wilcoxon signed-rank test.

Levels of nasal cytokines and chemokines were compared on day 3 in AA versus HC, using the Mann-Whitney test.

*P*-values associated with each test are shown, with significant values highlighted in pink.

## Table S7. Comparison of Nasal MLF Cytokine and Chemokine Levels in Allergic Asthmatics (AA) vs Healthy Controls (HC) on Day 0 and Day 4

| **Cytokine**  **Chemokine** | **HC**  **Day 0**  **Median** | **HC**  **Day 4**  **Median** | **AA**  **Day 0**  **Median** | **AA**  **Day 4**  **Median** | **HC**  **Day 0 vs 4**  ***P*** | **AA**  **Day 0-4**  ***P*** | **HC vs AA**  **Day 4**  ***P*** |
| --- | --- | --- | --- | --- | --- | --- | --- |
| **IL-1β** | 176.99 | 302.74 | 85.94 | 231.77 | 0.4131 | 0.6252 | 0.3738 |
| **IL-2** | 1.28 | 1.69 | 1.89 | 2.81 | 0.5195 | 0.1019 | 0.0727 |
| **IL-4** | 0 | 0 | 0.89 | 0.88 | 0.7500 | 0.9866 | 0.0351 |
| **IL-5** | 0 | 0 | 1.81 | 4.97 | 0.5625 | <0.0001 | 0.0012 |
| **IL-6** | 46.96 | 103.31 | 40.07 | 251.13 | 0.9658 | <0.0001 | 0.1013 |
| **IL-10** | 0 | 0 | 0 | 0.75 | 0.6250 | 0.0136 | 0.1038 |
| **IL-12p40** | 10.80 | 12.49 | 11.66 | 13.81 | 0.4648 | 0.4624 | 1.0000 |
| **IL-12p70** | 0 | 0 | 0 | 0.64 | 0.5000 | 0.4951 | 0.0982 |
| **IL-13** | 0 | 0 | 1.95 | 3.75 | 1.0000 | 0.0006 | 0.0004 |
| **IL-15** | 0.87 | 1.16 | 1.67 | 2.71 | 0.9658 | 0.0088 | 0.0511 |
| **IL-16** | 1409.01 | 700.96 | 1737.30 | 1746.44 | 0.8984 | 0.2079 | 0.2295 |
| **IL-17** | 2.23 | 2.91 | 4.86 | 4.91 | 1.0000 | 0.3719 | 0.1555 |
| **IL-18** | 509.79 | 734.38 | 418.36 | 229.19 | 0.5771 | 0.1549 | 0.0156 |
| **IL-25** | 0 | 0 | 0 | 0 | 0.5000 | 0.3804 | 0.2339 |
| **IL-33** | 45.46 | 40.74 | 26.52 | 67.81 | 0.3652 | 0.0054 | 0.6066 |
| **IFN-γ** | 0 | 0.85 | 0 | 3.078 | 0.5703 | <0.0001 | 0.0724 |
| **IFN-β** | 0 | 0 | 0 | 0 | . | 0.1250 | 0.2026 |
| **IFN-λ (IL-29)** | 0 | 0 | 0 | 0 | 1.0000 | 0.2114 | 0.0324 |
| **GM-CSF** | 0.89 | 0.78 | 0.86 | 1.09 | 0.9102 | 0.3669 | 0.7747 |
| **TNF-α** | 1.17 | 1.66 | 1.25 | 2.74 | 0.4961 | 0.0003 | 0.4620 |
| **TSLP** | 0 | 0 | 0 | 0 | 1.0000 | 0.2500 | 0.8659 |
| **CCL2/MCP-1** | 40.00 | 21.55 | 131.52 | 153.72 | 0.4648 | 0.1618 | 0.0409 |
| **CCL3/MIP-1α** | 9.675 | 11.49 | 9.32 | 12.38 | 0.2754 | 0.0155 | 0.6849 |
| **CCL4/MIP-1β** | 26.21 | 38.61 | 43.35 | 128.80 | 0.3203 | <0.0001 | 0.1301 |
| **CCL5/RANTES** | 18.55 | 26.10 | 19.90 | 27.95 | 0.4648 | 0.0069 | 0.5428 |
| **CCL11/eotaxin** | 0 | 0 | 16.13 | 19.53 | 0.3750 | 0.2688 | 0.0328 |
| **CCL13/MCP-4** | 0 | 0 | 3.84 | 3.99 | 0.8125 | 0.5392 | 0.0413 |
| **CCL17 /TARC** | 8.18 | 8.79 | 17.08 | 21.18 | 0.2061 | 0.0923 | 0.0156 |
| **CCL20/MIP-3α** | 3.78 | 4.57 | 5.76 | 7.59 | 0.9102 | 0.0071 | 0.1744 |
| **CCL22 /MDC** | 15.68 | 18.24 | 25.20 | 41.33 | 0.0830 | 0.0038 | 0.0475 |
| **CCL26/eotaxin-3** | 121.10 | 63.46 | 153.86 | 132.65 | 0.0244 | 0.2432 | 0.0185 |
| **CXCL8/IL-8** | 3171.24 | 2797.90 | 2662.59 | 2834.42 | 0.6377 | 0.9470 | 0.8883 |
| **CXCL10/ IP10** | 945.72 | 2097.34 | 1966.10 | 3614.59 | 0.2783 | 0.0014 | 0.0325 |
| **CXCL11 /ITAC** | 14.51 | 87.76 | 39.28 | 681.20 | 0.2402 | <0.0001 | 0.0278 |

HC=healthy controls, AA=allergic asthmatics

Levels of nasal cytokines and chemokines were compared in HC from day 0 to day 4 (n=11) and in AA from day 0 to day 4 (n=28).

Samples with values under lower limit of detection (LLD) were given a value of 0 pg/ml.

Paired samples were analysed by the Wilcoxon signed-rank test.

Levels of nasal cytokines and chemokines were compared on day 4 in AA versus HC, using the Mann-Whitney test.

*P*-values associated with each test are shown, with significant values highlighted in pink/blue.

Note that IL-18 levels are decreased in AA compared to HC after RV infection and CCL26/eotaxin-3 was decreased on day 4 compared to baseline in HC highlighted in blue.

## Table S8. Comparison of Nasal MLF Cytokine and Chemokine Levels in Allergic Asthmatics (AA) vs Healthy Controls (HC) on Day 0 and Day 5

| **Cytokine**  **Chemokine** | **HC**  **Day 0**  **Median** | **HC**  **Day 5**  **Median** | **AA**  **Day 0**  **Median** | **AA**  **Day 5**  **Median** | **HC**  **Day 0 vs 5**  ***P*** | **AA**  **Day 0 vs 5**  ***P*** | **HC vs AA**  **Day 5**  ***P*** |
| --- | --- | --- | --- | --- | --- | --- | --- |
| **IL-1β** | 176.99 | 157.78 | 85.94 | 154.31 | 1.0000 | 0.5189 | 0.7669 |
| **IL-2** | 1.28 | 1.77 | 1.89 | 1.89 | 0.9219 | 0.9442 | 0.4824 |
| **IL-4** | 0 | 0 | 0.89 | 0 | 1.0000 | 0.0484 | 0.9111 |
| **IL-5** | 0 | 0 | 1.81 | 3.80 | 0.8438 | 0.0002 | 0.0004 |
| **IL-6** | 46.96 | 103.44 | 40.07 | 323.81 | 0.6377 | <.0001 | 0.0185 |
| **IL-10** | 0 | 0 | 0 | 0.65 | 1.0000 | 0.0079 | 0.1790 |
| **IL-12p40** | 10.80 | 13.66 | 11.66 | 12.10 | 0.8311 | 0.9823 | 0.5637 |
| **IL-12p70** | 0 | 0 | 0 | 0 | 0.5000 | 0.2524 | 0.7194 |
| **IL-13** | 0 | 0 | 1.95 | 1.80 | 0.1875 | 0.0604 | <.0001 |
| **IL-15** | 0.87 | 1.91 | 1.67 | 2.96 | 0.2783 | 0.0002 | 0.1013 |
| **IL-16** | 1409.01 | 655.92 | 1737.30 | 1775.18 | 0.6377 | 0.4488 | 0.6964 |
| **IL-17** | 2.23 | 2.14 | 4.86 | 3.66 | 0.4961 | 0.6896 | 0.3253 |
| **IL-18** | 509.79 | 402.01 | 418.36 | 441.56 | 0.4131 | 0.6571 | 0.8883 |
| **IL-25** | 0 | 0 | 0 | 0 | 1.0000 | 0.3396 | 0.0436 |
| **IL-33** | 45.46 | 63.54 | 26.52 | 51.17 | 0.4648 | 0.0022 | 0.8883 |
| **IFN-γ** | 0 | 0.68 | 0 | 1.67 | 0.7422 | 0.0004 | 0.1365 |
| **IFN-β** | 0 | 0 | 0 | 0 | . | 0.7500 | 0.2799 |
| **IFN-λ (IL-29)** | 0 | 0 | 0 | 0 | 1.0000 | 0.8999 | 0.0656 |
| **GM-CSF** | 0.89 | 0.91 | 0.86 | 1.00 | 0.5703 | 0.0883 | 0.7157 |
| **TNF-α** | 1.17 | 1.31 | 1.25 | 2.46 | 0.2031 | <.0001 | 0.2462 |
| **TSLP** | 0 | 0 | 0 | 0 | 1.0000 | 0.4375 | 0.8887 |
| **CCL2/MCP-1** | 40.00 | 61.74 | 131.52 | 222.68 | 0.0186 | 0.0510 | 0.0950 |
| **CCL3/MIP-1α** | 9.675 | 10.12 | 9.32 | 10.65 | 0.9658 | 0.1459 | 0.5957 |
| **CCL4/MIP-1β** | 26.21 | 34.73 | 43.35 | 177.18 | 0.3223 | <.0001 | 0.0218 |
| **CCL5/RANTES** | 18.55 | 30.36 | 19.90 | 36.66 | 0.5771 | 0.0004 | 0.2062 |
| **CCL11/eotaxin** | 0 | 0 | 16.13 | 19.22 | 0.1250 | 0.8801 | 0.1351 |
| **CCL13/MCP-4** | 0 | 2.48 | 3.84 | 4.01 | 0.5625 | 0.8464 | 0.0485 |
| **CCL17 /TARC** | 8.18 | 9.57 | 17.08 | 17.53 | 0.0977 | 0.3599 | 0.0185 |
| **CCL20/MIP-3α** | 3.78 | 4.03 | 5.76 | 6.17 | 0.6523 | 0.3585 | 0.4620 |
| **CCL22 /MDC** | 15.68 | 19.26 | 25.20 | 25.51 | 0.1230 | 0.7227 | 0.3104 |
| **CCL26/eotaxin-3** | 121.10 | 126.18 | 153.86 | 165.57 | 0.3203 | 0.1178 | 0.0833 |
| **CXCL8/IL-8** | 3171.24 | 3552.79 | 2662.59 | 2999.53 | 0.9658 | 0.5634 | 0.7908 |
| **CXCL10/ IP10** | 945.72 | 2914.20 | 1966.10 | 3768.47 | 0.1230 | 0.0012 | 0.0950 |
| **CXCL11 /ITAC** | 14.51 | 285.14 | 39.28 | 627.07 | 0.1748 | <.0001 | 0.0325 |

HC=healthy controls, AA=allergic asthmatics

Levels of nasal cytokines and chemokines were compared in HC from day 0 to day 5 (n=11) and in AA from day 0 to day 5 (n=28).

Samples with values under lower limit of detection (LLD) were given a value of 0 pg/ml.

Paired samples were analysed by the Wilcoxon signed-rank test.

Levels of nasal cytokines and chemokines were compared on day 5 in AA versus HC, using the Mann-Whitney test.

*P*-values associated with each test are shown, with significant values highlighted in pink/blue.

Note that IL-4 levels are decreased after RV infection in asthma highlighted in blue.

## Table S9. Comparison of Nasal MLF Cytokine and Chemokine Levels in Allergic Asthmatics (AA) vs Healthy Controls (HC) on Day 0 and Day 7

| **Cytokine**  **Chemokine** | **HC**  **Day 0**  **Median** | **HC**  **Day 7**  **Median** | **AA**  **Day 0**  **Median** | **AA**  **Day 7**  **Median** | **HC**  **Day 0 vs 7**  ***P*** | **AA**  **Day 0 vs 7**  ***P*** | **HC vs AA**  **Day 7**  ***P*** |
| --- | --- | --- | --- | --- | --- | --- | --- |
| **IL-1β** | 176.99 | 102.07 | 85.94 | 63.19 | 0.5771 | 0.6252 | 0.5637 |
| **IL-2** | 1.28 | 1.26 | 1.89 | 1.34 | 0.3203 | 0.3366 | 0.4632 |
| **IL-4** | 0 | 0 | 0.89 | 0 | 0.5000 | 0.0298 | 0.4024 |
| **IL-5** | 0 | 0 | 1.81 | 2.52 | 0.6250 | 0.2566 | 0.0476 |
| **IL-6** | 46.96 | 75.29 | 40.07 | 164.56 | 0.8984 | <.0001 | 0.0549 |
| **IL-10** | 0 | 0 | 0 | 0 | 0.7500 | 0.1202 | 0.2410 |
| **IL-12p40** | 10.80 | 13.72 | 11.66 | 10.25 | 0.3223 | 1.0000 | 0.2958 |
| **IL-12p70** | 0 | 0 | 0 | 0 | 1.0000 | 0.1205 | 0.5540 |
| **IL-13** | 0 | 0 | 1.95 | 1.92 | 0.4375 | 0.9508 | 0.0256 |
| **IL-15** | 0.87 | 1.44 | 1.67 | 2.69 | 0.1475 | 0.0008 | 0.0379 |
| **IL-16** | 1409.01 | 697.65 | 1737.30 | 512.05 | 0.2402 | 0.2722 | 0.9627 |
| **IL-17** | 2.23 | 1.65 | 4.86 | 2.30 | 0.3008 | 0.1689 | 0.4257 |
| **IL-18** | 509.79 | 361.33 | 418.36 | 301.32 | 0.0830 | 0.5045 | 0.5022 |
| **IL-25** | 0 | 0 | 0 | 10.83 | 0.5000 | 0.0730 | 0.0789 |
| **IL-33** | 45.46 | 31.11 | 26.52 | 26.21 | 0.4648 | 0.8593 | 0.7908 |
| **IFN-γ** | 0 | 0 | 0 | 1.64 | 1.0000 | 0.0019 | 0.0601 |
| **IFN-β** | 0 | 0 | 0 | 0 | . | 1.0000 | 0.3913 |
| **IFN-λ (IL-29)** | 0 | 0 | 0 | 0 | 1.0000 | 0.8926 | 0.0981 |
| **GM-CSF** | 0.89 | 0.86 | 0.86 | 0.79 | 0.3594 | 0.5217 | 0.8096 |
| **TNF-α** | 1.17 | 1.28 | 1.25 | 1.68 | 1.0000 | 0.0227 | 0.4799 |
| **TSLP** | 0 | 0 | 0 | 0 | 1.0000 | 1.0000 | 0.2799 |
| **CCL2/MCP-1** | 40.00 | 86.20 | 131.52 | 179.92 | 0.2061 | 0.3033 | 0.0351 |
| **CCL3/MIP-1α** | 9.675 | 9.41 | 9.32 | 8.82 | 0.7646 | 0.4355 | 0.6509 |
| **CCL4/MIP-1β** | 26.21 | 53.64 | 43.35 | 108.76 | 0.1309 | <.0001 | 0.0678 |
| **CCL5/RANTES** | 18.55 | 19.08 | 19.90 | 28.67 | 0.9658 | 0.0041 | 0.1467 |
| **CCL11/eotaxin** | 0 | 0 | 16.13 | 21.20 | 0.4375 | 0.3843 | 0.0277 |
| **CCL13/MCP-4** | 0 | 0 | 3.84 | 3.89 | 0.6250 | 0.9480 | 0.0087 |
| **CCL17 /TARC** | 8.18 | 10.30 | 17.08 | 23.76 | 0.0098 | 0.0403 | 0.0201 |
| **CCL20/MIP-3α** | 3.78 | 4.46 | 5.76 | 7.38 | 1.0000 | 0.5209 | 0.6044 |
| **CCL22 /MDC** | 15.68 | 22.21 | 25.20 | 30.63 | 0.0010 | 0.2927 | 0.1224 |
| **CCL26/eotaxin-3** | 121.10 | 112.50 | 153.86 | 171.89 | 0.5195 | 0.5045 | 0.0325 |
| **CXCL8/IL-8** | 3171.24 | 3598.07 | 2662.59 | 2201.62 | 0.8311 | 0.8767 | 0.7197 |
| **CXCL10/ IP10** | 945.72 | 2455.91 | 1966.10 | 4186.21 | 0.3652 | <.0001 | 0.0014 |
| **CXCL11 /ITAC** | 14.51 | 39.17 | 39.28 | 632.06 | 0.2402 | <.0001 | 0.0156 |

HC=healthy controls, AA=allergic asthmatics

Levels of nasal cytokines and chemokines were compared in HC from day 0 to day 7 (n=11) and in AA from day 0 to day 7 (n=28).

Samples with values under lower limit of detection (LLD) were given a value of 0 pg/ml.

Paired samples were analysed by the Wilcoxon signed-rank test.

Levels of nasal cytokines and chemokines were compared on day 7 in AA versus HC, using the Mann-Whitney test.

*P*-values associated with each test are shown, with significant values highlighted in pink/blue.

Note that IL-4 levels are decreased after RV infection in asthma highlighted in blue.

## Table S10. Nasosorption Changes in Cytokines and Chemokines from Baseline for Allergic Asthmatics (AA) and Healthy Controls (HC)

| **Cytokine**  **Chemokine** |  | **Change**  **Day 0-2**  **(pg/ml)** | **Change Day 0-3**  **(pg/ml)** | **Change Day 0-4**  **(pg/ml)** | **Change Day 0-5**  **(pg/ml)** | **Change Day 0-7 (pg/ml)** |
| --- | --- | --- | --- | --- | --- | --- |
| **IL-1β** | HC | 4.896143 | 83.35645 | 48.74985 | 3.386767 | -25.4767 |
|  | AA | -27.6929 | 10.18896 | 13.03852 | 6.840553 | 2.681954 |
|  | *P* | 0.2482 | 0.2886 | 0.5532 | 0.7550 | 0.2747 |
| **IL-2** | HC | 0.179836 | -0.04717 | 0.153331 | -0.14878 | -0.37183 |
|  | AA | 0.530482 | 1.497149 | 0.542094 | -0.12513 | -0.18408 |
|  | *P* | 0.2612 | 0.0919 | 0.8515 | 0.8149 | 0.9751 |
| **IL-4** | HC | 0 | 0 | 0 | 0 | 0 |
|  | AA | 0 | 0.045869 | 0 | -0.47024 | -0.52188 |
|  | *P* | 0.7724 | 0.4472 | 0.9232 | 0.1230 | 0.4083 |
| **IL-5** | HC | 0 | 0 | 0 | 0 | 0 |
|  | AA | 0.529487 | 3.07483 | 3.582823 | 2.343086 | 0 |
|  | *P* | 0.2707 | 0.0695 | 0.0154 | 0.0151 | 0.9874 |
| **IL-6** | HC | -5.5936 | 37.54536 | 16.3993 | 36.58582 | 0.854443 |
|  | AA | 11.58811 | 252.0008 | 160.117 | 272.0834 | 86.38284 |
|  | *P* | 0.1186 | 0.1602 | 0.0267 | 0.0041 | 0.0125 |
| **IL-10** | HC | 0 | 0 | 0 | 0 | 0 |
|  | AA | 0 | 0.413497 | 0.00529 | 0.032573 | 0 |
|  | *P* | 0.0633 | 0.4722 | 0.3541 | 0.0588 | 0.8944 |
| **IL-12p40** | HC | 2.781454 | 1.953011 | 0.550307 | -1.54446 | 0.672309 |
|  | AA | 0.962941 | 0.949998 | 0.755783 | 0.268774 | 1.457239 |
|  | *P* | 0.2235 | 0.3491 | 0.6849 | 0.9007 | 0.4538 |
| **IL-12p70** | HC | 0 | 0 | 0 | 0 | 0 |
|  | AA | 0 | 0 | 0 | 0 | 0 |
|  | *P* | 0.4207 | 0.6386 | 0.8954 | 0.0960 | 0.1847 |
| **IL-13** | HC | 0 | 0 | 0 | 0 | 0 |
|  | AA | 0.005797 | 0.967011 | 1.51818 | 0.158509 | -0.13758 |
|  | *P* | 0.7782 | 0.0254 | 0.0122 | 0.0644 | 0.9750 |
| **IL-15** | HC | 0 | 0.146294 | 0.082772 | 0.323933 | 0.294844 |
|  | AA | 0.105238 | 0.679296 | 0.899285 | 1.285344 | 0.84043 |
|  | *P* | 0.4729 | 0.3654 | 0.1796 | 0.1511 | 0.4729 |
| **IL-16** | HC | -5.56498 | 179.7775 | 58.77731 | 106.6137 | -36.7486 |
|  | AA | -6.13779 | -44.8073 | 294.5402 | -28.8644 | -138.067 |
|  | *P* | 0.6397 | 0.1511 | 0.4923 | 0.3994 | 0.9254 |
| **IL-17** | HC | 0.685595 | 0.795957 | 0 | 0 | -0.71729 |
|  | AA | 0.449371 | 0.502469 | 0.14537 | -0.61944 | -1.96903 |
|  | *P* | 0.7550 | 0.8760 | 1.0000 | 0.8028 | 0.4538 |

| **Cytokine**  **Chemokine** |  | **Change**  **Day 0-2**  **(pg/ml)** | **Change Day 0-3**  **(pg/ml)** | **Change Day 0-4**  **(pg/ml)** | **Change Day 0-5**  **(pg/ml)** | **Change Day 0-7 (pg/ml)** |
| --- | --- | --- | --- | --- | --- | --- |
| **IL-18** | HC | -95.5395 | -304.505 | 50.15111 | -185.183 | -133.31 |
|  | AA | -165.175 | -33.2546 | -41.725 | -7.20795 | -39.9773 |
|  | *P* | 0.6622 | 0.3333 | 0.2235 | 0.5957 | 0.4923 |
| **IL-25** | HC | 0 | 0 | 0 | 0 | 0 |
|  | AA | 0 | 0 | 0 | 0 | 0 |
|  | *P* | 0.4984 | 0.8030 | 0.9710 | 0.3445 | 0.4801 |
| **IL-33** | HC | -14.6841 | -18.3073 | -7.23837 | -1.27201 | -1.26717 |
|  | AA | 2.348056 | 4.358352 | 13.58173 | 24.52332 | -3.92516 |
|  | *P* | 0.0080 | 0.1697 | 0.0530 | 0.0981 | 0.5122 |
| **IFN-γ** | HC | 0 | 0 | 0.335097 | 0 | 0 |
|  | AA | 1.027082 | 4.802329 | 2.440481 | 1.446941 | 0.91897 |
|  | *P* | 0.1085 | 0.1031 | 0.0492 | 0.1111 | 0.1380 |
| **IFN-β** | HC | 0 | 0 | 0 | 0 | 0 |
|  | AA | 0 | 0 | 0 | 0 | 0 |
|  | *P* | 1.0000 | 0.0726 | 0.1923 | 0.7103 | 1.0000 |
| **IFN-λ (IL-29)** | HC | 0 | 0 | 0 | 0 | 0 |
|  | AA | 23.35685 | 0 | 0 | 0 | 0 |
|  | *P* | 0.0240 | 0.6627 | 0.2623 | 0.5868 | 0.9008 |
| **GM-CSF** | HC | 0 | 0.078418 | -0.11085 | 0 | -0.03188 |
|  | AA | 0 | 0 | 0 | 0.312559 | 0 |
|  | *P* | 0.6369 | 0.9126 | 0.3471 | 0.1777 | 0.2442 |
| **TNF-α** | HC | 0.276462 | 0.623452 | 0 | 0.052683 | 0 |
|  | AA | 0.481643 | 0.885057 | 1.25976 | 1.31462 | 0.233661 |
|  | *P* | 0.8023 | 0.7788 | 0.4350 | 0.1251 | 0.2873 |
| **TSLP** | HC | 0 | 0 | 0 | 0 | 0 |
|  | AA | 0 | 0 | 0 | 0 | 0 |
|  | *P* | 0.4697 | 0.1640 | 0.2166 | 0.7976 | 0.7279 |
| **CCL2/MCP-1** | HC | -0.10858 | 27.38076 | 10.77019 | 33.35388 | 4.811063 |
|  | AA | 29.34434 | 27.22592 | 28.1829 | 50.69646 | 54.65729 |
|  | *P* | 0.1262 | 0.6622 | 1.0000 | 0.9751 | 0.8028 |
| **CCL3/MIP-1α** | HC | 1.321043 | 1.985938 | 0.352511 | -0.64248 | 0.031883 |
|  | AA | 0.211489 | 0.56818 | 2.39997 | 1.663213 | 0.472621 |
|  | *P* | 0.4538 | 0.6849 | 0.9627 | 0.4538 | 0.9007 |
| **CCL4/MIP-1β** | HC | 7.806604 | 27.65412 | 15.10235 | 11.99281 | 2.982577 |
|  | AA | 8.906742 | 28.7341 | 78.37452 | 101.5535 | 42.1095 |
|  | *P* | 0.9502 | 0.5743 | 0.2886 | 0.0209 | 0.1046 |

| **Cytokine**  **Chemokine** |  | **Change**  **Day 0-2**  **(pg/ml)** | **Change Day 0-3**  **(pg/ml)** | **Change Day 0-4**  **(pg/ml)** | **Change Day 0-5**  **(pg/ml)** | **Change Day 0-7 (pg/ml)** |
| --- | --- | --- | --- | --- | --- | --- |
| **CCL5/RANTES** | HC | 0.699062 | -4.01569 | 14.6175 | -4.31416 | 2.955898 |
|  | AA | 3.130231 | 10.4418 | 13.6546 | 14.02832 | 14.07947 |
|  | *P* | 0.2007 | 0.0530 | 0.4729 | 0.0192 | 0.0493 |
| **CCL11/eotaxin** | HC | 0 | 0 | 0 | 0 | 0 |
|  | AA | 0 | 10.24169 | 0 | 0 | 0 |
|  | *P* | 0.7276 | 0.9249 | 0.9363 | 0.2657 | 0.3752 |
| **CCL13/MCP-4** | HC | 0 | 0 | 0 | 0 | 0 |
|  | AA | -0.20073 | 0 | 0 | -0.309 | 0 |
|  | *P* | 0.2523 | 0.2887 | 0.7276 | 0.3462 | 0.6940 |
| **CCL17/TARC** | HC | 2.192012 | 3.940709 | 1.081471 | 1.387648 | 2.740467 |
|  | AA | 2.12661 | 3.627001 | 1.759673 | 1.113497 | 4.536954 |
|  | *P* | 0.9627 | 0.9502 | 1.0000 | 0.8271 | 0.8515 |
| **CCL20/MIP-3α** | HC | 0 | 1.89012 | 0 | 0 | 0 |
|  | AA | 1.630327 | 2.124847 | 1.388556 | 0.268354 | 0 |
|  | *P* | 0.0654 | 0.8759 | 0.1600 | 0.7549 | 0.7547 |
| **CCL22/MDC** | HC | 1.720215 | 3.72136 | 1.525747 | 3.812248 | 2.389981 |
|  | AA | 5.574382 | 9.909228 | 4.953472 | -0.50362 | 1.523106 |
|  | *P* | 0.0805 | 0.5532 | 0.2747 | 0.1899 | 0.3031 |
| **CCL26/eotaxin-3** | HC | -7.14875 | 3.667789 | -44.6878 | 5.048998 | -8.59603 |
|  | AA | -13.6911 | -32.657 | -38.2558 | 8.164727 | -4.93429 |
|  | *P* | 0.7080 | 0.5743 | 0.8760 | 0.8271 | 0.9751 |
| **CXCL8/IL-8** | HC | 237.2756 | 1319.377 | -371.192 | -52.3462 | -156.221 |
|  | AA | -69.3893 | -248.233 | 101.7815 | 66.84841 | 81.58809 |
|  | *P* | 0.2886 | 0.1341 | 0.7788 | 0.7550 | 0.9751 |
| **CXCL10/IP10** | HC | 34.94205 | 263.5196 | 372.6947 | 884.5542 | 882.4207 |
|  | AA | 523.7666 | 2149.945 | 1712.507 | 1987.082 | 2351.025 |
|  | *P* | 0.3031 | 0.1424 | 0.1341 | 0.2747 | 0.0096 |
| **CXCL11/ITAC** | HC | 4.305447 | 9.889353 | 21.58144 | 14.28504 | 28.07448 |
|  | AA | 23.44392 | 577.0503 | 667.0964 | 612.9653 | 333.8068 |
|  | *P* | 0.2886 | 0.1602 | 0.0753 | 0.0805 | 0.0073 |

The change in values from baseline (pg/ml) for each individual were first calculated.

The median for changes on days 2-7 from baseline were then calculated for HC and AA.

*P* values were calculated by Mann-Whitney test for HC versus AA, significance where *P*<0.05 highlighted in pink.

## Table S11. Receiver Operating Characteristics (ROC) for Nasal Cytokines and Chemokines on Day 0 and Day 4 in Relation to Allergic Asthmatics (AA) and Healthy Controls (HC)

| **Cytokine**  **Chemokine** | **Area under Curve (AUC)** | | **Optimal Performance Point (OPP )**  **Sensitivity** | | **OPP**  **1-Specificity** | |
| --- | --- | --- | --- | --- | --- | --- |
|  | **Day 0** | **Day 4** | **Day 0** | **Day 4** | **Day 0** | **Day 4** |
| **IFN-γ** | 0.50 | 0.50 | 0 | 0 | 0 | 0 |
| **IFN-λ (IL-29)** | 0.50 | 0.50 | 0 | 0 | 0 | 0 |
| **CXCL11 (ITAC)** | 0.50 | 0.50 | 0 | 0 | 0 | 0 |
| **CXCL10 (IP10)** | 0.50 | 0.50 | 0 | 0 | 0 | 0 |
| **IL-6** | 0.50 | 0.50 | 0 | 0 | 0 | 0 |
| **IL-15** | 0.50 | 0.50 | 0 | 0 | 0 | 0 |
| **CCL4 (MIP-1β)** | 0.50 | 0.50 | 0 | 0 | 0 | 0 |
| **IL-4** | 0.66 | 0.68 | 0.79 | 0.80 | 0.70 | 0.70 |
| **IL-5** | 0.73 | 0.78 | 0.85 | 0.85 | 0.67 | 0.67 |
| **IL-13** | 0.75 | 0.84 | 0.68 | 0.85 | 0.85 | 0.82 |
| **CCL17 (TARC)** | 0.50 | 0.50 | 0 | 0 | 0 | 0 |
| **CCL22 (MDC)** | 0.50 | 0.67 | 0 | 0.20 | 0 | 0.95 |
| **CCL11 (eotaxin)** | 0.50 | 0.50 | 0 | 0 | 0 | 0 |

The Receiver Operating Characteristic (ROC) curve analyses were performed on normal transformed data using the Monte Carlo cross-validation method, which splits the data into training and test sets, and used a predictive model based on linear discriminate analysis (LDA).

The prediction accuracy was calculated using nested cross-validation to measure the ROC area under the curve (AUC)

The optimum performance point (OPP) is that point on the ROC curve with a maximal sum of sensitivity and specificity.

The line of no discrimination has an AUC of 0.50, sensitivity of 0 and specificity of 1

.

## Table S12. Comparison of Bronchial MLF Cytokine and Chemokine Levels in Allergic Asthmatics (AA) vs Healthy Controls (HC) at Baseline (BL) and Day 4

| **Cytokine**  **Chemokine** | **HC**  **BL**  **Median** | **HC**  **Day 4**  **Median** | **AA**  **BL**  **Median** | **AA**  **Day 4**  **Median** | **HC**  **BL-Day 4**  ***P*** | **AA**  **BL-Day 4**  ***P*** | **HC- AA**  **BL**  ***P*** | **HC-AA**  **Day 4**  ***P*** |
| --- | --- | --- | --- | --- | --- | --- | --- | --- |
| **IL-1β** | 11.4638 | 13.8135 | 5.3364 | 9.7229 | 0.5566 | 0.5155 | 0.0280 | 0.3908 |
| **IL-2** | 2.0255 | 1.8262 | 1.9928 | 2.1073 | 0.9219 | 0.8599 | 0.9726 | 0.7014 |
| **IL-4** | 0 | 0 | 0 | 0 | 0.4375 | 0.2961 | 0.5708 | 0.0527 |
| **IL-5** | 0 | 0 | 0.6462 | 1.0916 | 0.6250 | 0.0443 | 0.0872 | 0.0547 |
| **IL-6** | 51.0736 | 83.4226 | 28.1646 | 51.5745 | 0.4922 | 0.2566 | 0.0924 | 0.1826 |
| **IL-10** | 0.0000 | 0.0000 | 0.0000 | 0.0000 | 0.8750 | 0.0391 | 0.8119 | 0.6898 |
| **IL-12p40** | 25.7474 | 24.1167 | 32.2273 | 35.8801 | 0.3750 | 0.1481 | 0.9726 | 0.1042 |
| **IL-12p70** | 0.6161 | 0 | 0 | 0 | 0.1250 | 1.0000 | 0.0455 | 0.6313 |
| **IL-13** | 0 | 0 | 0.6091 | 0.7093 | 1.0000 | 0.2412 | 0.0715 | 0.0072 |
| **IL-15** | 0.9231 | 1.0996 | 0.7958 | 1.1660 | 0.8203 | 0.0258 | 0.9444 | 0.2968 |
| **IL-16** | 3876.0530 | 2309.5940 | 2564.6620 | 2851.1790 | 0.2324 | 0.4237 | 0.4296 | 0.7014 |
| **IL-17** | 5.4323 | 5.1663 | 3.4875 | 6.3667 | 0.7695 | 0.0618 | 0.0741 | 0.6481 |
| **IL-18** | 103.2006 | 135.3550 | 148.4076 | 136.7662 | 0.4922 | 0.8599 | 0.3538 | 0.7287 |
| **IL-25** | 0 | 0 | 0 | 0 | 1.0000 | 0.6250 | 0.8531 | 0.4961 |
| **IL-33** | 1500.0590 | 2354.0140 | 1826.2570 | 1835.6550 | 0.2754 | 0.8139 | 0.2294 | 0.5468 |
| **IFN-γ** | 1.0358 | 2.0573 | 0.7309 | 2.1855 | 0.8457 | 0.0028 | 0.0699 | 0.5943 |
| **IFN-β** | 0 | 0 | 0 | 0 | 1.0000 | 0.3750 | 0.6038 | 0.1416 |
| **IFN-λ (IL-29)** | 0 | 0 | 0 | 0 | 1.0000 | 0.4688 | 0.3956 | 0.4202 |
| **GM-CSF** | 1.7320 | 1.7857 | 1.3357 | 1.7368 | 0.4316 | 0.0760 | 0.0500 | 0.9563 |
| **TNF-α** | 0.7229 | 0.6344 | 0 | 0.8540 | 0.4688 | 0.0004 | 0.3960 | 0.3669 |
| **TSLP** | 0 | 0 | 0 | 0 | 1.0000 | 0.0000 | 0.0341 | 1.0000 |
| **CCL2/MCP-1** | 105.5507 | 159.4356 | 88.8683 | 106.7044 | 0.8457 | 0.6161 | 0.2871 | 0.0895 |
| **CCL3/MIP-1α** | 7.5087 | 7.7019 | 7.1681 | 7.9039 | 0.6953 | 0.1014 | 0.6553 | 0.7287 |
| **CCL4/MIP-1β** | 55.8828 | 66.0822 | 63.0716 | 85.5825 | 0.6250 | 0.3264 | 0.8100 | 0.8695 |
| **CCL5/RANTES** | 2845.9840 | 1359.1360 | 2633.9200 | 2791.3690 | 0.3223 | 0.0717 | 0.7835 | 0.1207 |
| **CCL11/eotaxin** | 31.5469 | 20.6811 | 24.9000 | 22.0721 | 0.6250 | 0.4065 | 0.5028 | 0.5228 |
| **CCL13/MCP-4** | 3.2505 | 3.4146 | 3.6683 | 4.3198 | 0.7695 | 0.4392 | 0.3998 | 0.0407 |
| **CCL17 /TARC** | 26.6106 | 24.5073 | 24.0062 | 28.0433 | 1.0000 | 0.7017 | 0.3906 | 0.2654 |
| **CCL20/MIP-3α** | 87.1241 | 138.2148 | 60.2159 | 96.1384 | 0.8457 | 0.9063 | 0.1308 | 0.1492 |
| **CCL22 /MDC** | 18.2124 | 20.2596 | 22.6377 | 24.7257 | 0.7695 | 0.2198 | 0.9452 | 0.7563 |
| **CCL26/eotaxin-3** | 82.3311 | 65.2182 | 95.7596 | 99.7090 | 0.4922 | 0.6799 | 0.0993 | 0.0124 |
| **CXCL8/IL-8** | 535.7467 | 762.0608 | 568.8395 | 593.0681 | 0.8457 | 0.6799 | 0.3193 | 0.3518 |
| **CXCL10/ IP10** | 1016.5880 | 2277.7300 | 1011.6770 | 1998.7900 | 0.3750 | <0.0001 | 0.3193 | 0.7842 |
| **CXCL11 /ITAC** | 24.4738 | 77.7579 | 19.4380 | 119.7780 | 0.3223 | <0.0001 | 0.1805 | 0.5714 |

Pre = bronchosorption sample taken on day ~-14 before RV infection

Post = bronchosorption sample taken on day 4 after RV infection

AA = allergic asthma patient, HC = healthy non-allergic control subject

*P*-values were derived from the Mann-Whitney test (non-paired) in allergic asthmatics vs. healthy controls, within groups the Wilcoxon signed-rank test was used for paired samples..

Significant *P* values (<0.05) are shaded in pink/blue.

Note IL-1β was lower in AA than in HC at baseline.

Baseline bronchosorption samples were not obtained from 3 asthmatics; day 4 bronchosorption samples were not obtained from 3 asthmatic and 1 healthy subject. Therefore this data is based on n=11 HCs at baseline, n=10 HCs on day 4, and n=25 AA at baseline and on day 4.
